# Associations of sarcopenia, sarcopenia components and sarcopenic obesity with cancer incidence: A prospective cohort study of 414,094 participants in UK Biobank

**DOI:** 10.1002/ijc.35480

**Published:** 2025-05-21

**Authors:** Panagiotis Filis, Christos K. Papagiannopoulos, Georgios Markozannes, Christos V. Chalitsios, Ioannis Zerdes, Antonios Valachis, Christopher Papandreou, Sofia Christakoudi, Konstantinos K. Tsilidis

**Affiliations:** ^1^ Department of Hygiene and Epidemiology University of Ioannina School of Medicine Ioannina Greece; ^2^ Department of Medical Oncology University of Ioannina School of Medicine Ioannina Greece; ^3^ Department of Epidemiology and Biostatistics, School of Public Health Imperial College London London UK; ^4^ Department of Oncology‐Pathology Karolinska Institutet Stockholm Sweden; ^5^ Theme Cancer Karolinska University Hospital Stockholm Sweden; ^6^ Department of Oncology, Faculty of Medicine and Health Örebro University Örebro Sweden; ^7^ Institut d'Investigació Sanitària Pere Virgili (IISPV), NeuroÈpia Group Hospital Universitari Sant Joan de Reus Reus (Tarragona) Spain; ^8^ Department of Nutrition and Dietetics Sciences, School of Health Sciences Hellenic Mediterranean University (HMU) Siteia Greece; ^9^ Present address: Department of Oncology‐Pathology Karolinska Institutet Stockholm Sweden

**Keywords:** cancer, grip strength, muscle mass, sarcopenia, sarcopenic obesity

## Abstract

Sarcopenia is characterised by low grip strength, muscle quantity or quality, and physical performance. This study investigated the associations of sarcopenia and its components with cancer incidence. A prospective cohort study was conducted utilising data from the UK Biobank. Sarcopenia and its components were defined according to the European Working Group on Sarcopenia in Older People criteria (EWGSOP2 2019). Cox proportional hazard models adjusted for sociodemographic, lifestyle, and health‐related factors were performed. Overall, 63,379 out of 414,094 study participants had an incident diagnosis of cancer during a median follow‐up of 11.7 years. In total, 32,286 participants had probable sarcopenia and 934 confirmed/severe sarcopenia at recruitment. Combined probable, confirmed, and severe sarcopenia was associated with a higher risk of liver (hazard ratio [HR] = 1.65, 95% confidence interval [CI]: 1.17–2.33), haematological (HR = 1.22, 95% CI: 1.01–1.46), and colorectal cancer (HR = 1.21, 95% CI: 1.04–1.41) in males, but not in females. The components of sarcopenia were associated with a higher risk of several cancers, including low grip strength (with liver, haematological and colorectal cancer in males), low muscle mass index (oesophageal in females and oral cancer in males), and slow walking pace (liver and lung in males, lung and overall cancer in females). Compared to participants with non‐sarcopenic obesity, those with sarcopenic obesity had a higher risk of colorectal cancer in males (HR = 1.31, 95% CI: 1.03–1.68). Our study suggests that sarcopenia, sarcopenia components, and sarcopenic obesity can be associated with risk for several cancers, mainly of the gastrointestinal tract and in males. Thus, early identification of sarcopenia components may benefit cancer prevention.

## What's New?

Sarcopenia is highly prevalent and associated with adverse outcomes in cancer survivors. However, evidence regarding the role of sarcopenia in the general population as a potential risk factor for cancer development is scarce. This prospective cohort study investigated the association of sarcopenia with the incidence of 19 cancers in a Caucasian population, following the criteria of its latest formal definition. Sarcopenia, sarcopenia components and sarcopenic obesity were associated with risk for several cancers, mainly of the gastrointestinal tract and in males. Since sarcopenia is a manageable and treatable condition, early identification may benefit cancer prevention.

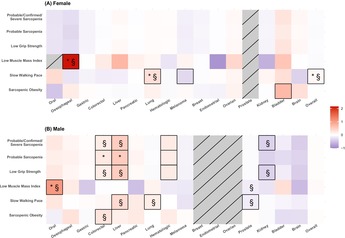

**Abbreviations**
AWGS: Asian Working Group on Sarcopenia
BIA: bioelectrical impedance analysis
BMI: body mass index
CI: confidence interval
EWGSOP2: European Working Group on Sarcopenia in Older People
FDR: false discovery rate
FNIH: Foundation for the National Institute of Health
HRT: hormone replacement therapy
HR: hazard ratio
ICD‐10: International Classification of Diseases, Tenth Revision
IQR: interquartile range
IWGS: International Working Group on Sarcopenia
kg: kilogram
MET: metabolic equivalent of task
MMI: muscle mass index
m: metre
NSAIDs: non‐steroidal anti‐inflammatory drugs
SD: standard deviation
SHBG: sex hormone binding globulin
TDI: Townsend deprivation index
UKB: UK Biobank

## 1 INTRODUCTION

Sarcopenia is an age‐related musculoskeletal disorder linked to a plethora of etiological and pathophysiological factors, such as anorexia, lack of physical activity, decline in nutritional intake, genetic causes, inflammatory pathway activation, mitochondrial dysfunction, loss of neuromuscular junctions, reduced satellite cell numbers, hypogonadism, insulin resistance, and poor blood flow to muscle.^1^, ^2^ From the introduction of the term in 1989,^3^ to its recognition as a muscle disease in the ICD‐10‐CM (International Classification of Diseases, Tenth Revision, Clinical Modification) in 2016 and beyond,^1^ the evolution of sarcopenia characterisation went through various criteria. In its updated definition established by the European Working Group on Sarcopenia in Older People (EWGSOP2), the principal determinant has shifted from low muscle mass to low muscle strength to facilitate prompt diagnosis of sarcopenia in clinical practice.^4^ Another main difference compared to the initial definition (EWGSOP 2010),^5^ is that sarcopenia characterisation is no longer restricted to older populations.^4^ Although the definition was endorsed by a range of international scientific societies, such as the Asian Working Group on Sarcopenia (AWGS) with population‐specific cut‐offs, other definitions with similar criteria also exist, including the International Working Group on Sarcopenia (IWGS) and the Foundation for the National Institute of Health (FNIH).^6^

The prevalence of this muscle condition in the general population varies greatly between definitions and the geographical origin of the studied populations.^7^, ^8^ A recent systematic review found that the summary prevalence for all sarcopenia definitions ranged from 8% to 36% for individuals younger than 60 years and from 10% to 27% for individuals older than 60 years.^8^ When considering only the newest EWGSOP2 definition, the prevalence of sarcopenia was 10% (ranging from 2% to 17%) for populations older than 60 years, whereas no data exist for younger individuals.^8^ These numbers could be considered alarming since sarcopenia has been associated with detrimental effects on quality of life domains, poor physical performance and depression.^9^ Furthermore, sarcopenia has been associated with increased risk for a variety of detrimental health outcomes, including longer hospitalisation and postoperative complications in patients, as well as falls and fractures, cognitive impairment, metabolic disorders and mortality in the general population.^10^

In cancer survivors specifically, sarcopenia is also being studied extensively due to its high prevalence and association with adverse outcomes.^11^ Despite the increasing interest in the prognostic value of sarcopenia following a cancer diagnosis, its potential as a risk factor for cancer development in the general population remains to be clarified. Currently, only two studies in Asian populations have investigated sarcopenia and risk for cancer. A propensity score‐matched Asian population‐based cohort study observed a higher risk of numerous cancer sites for sarcopenic individuals,^12^ whereas in a Korean cohort sarcopenia was linked with higher gastric cancer incidence.^13^ The mechanistic association of sarcopenia with cancer could be attributed to DNA damage over time as a consequence of the chronic inflammatory state that characterises sarcopenia.^12^, ^14^ Existing data support further and more refined research, based on the newly introduced definitions, on sarcopenia as a risk factor for cancer and highlight the requirement of including Western populations.

Since sarcopenia constitutes a preventable and treatable condition,^15^ this prospective cohort study aimed to investigate the associations of sarcopenia and its components with the incidence of 19 cancers in a Caucasian population, using data from the UK Biobank (UKB).

## 2 MATERIALS AND METHODS

### 2.1 Data Sources

UKB is a multicentre prospective cohort study, including over half a million participants aged 37–70 years old living in the UK.^16^ Recruitment was conducted in 22 assessment centres across the UK between 2006 and 2010.

### 2.2 Sarcopenia & its Components

Sarcopenia was defined following the EWGSOP2 recommendation.^4^ The sarcopenia continuum was classified into three categories: probable sarcopenia, confirmed sarcopenia, and severe sarcopenia.

Probable sarcopenia was defined as low hand grip strength, with cut‐off values of <27 kg for males and <16 kg for females.^4^ Hand grip strength was assessed using a Jamar J00105 hydraulic hand dynamometer, and the mean of the right‐ and left‐hand values, expressed in kilogram (kg), was used in the analysis, as reported elsewhere.^17^

Sarcopenia was confirmed when participants had both low grip strength and low muscle mass index (MMI). The established sarcopenia cut‐off points for MMI values were <7 kg/m^2^ for males and <5.5 kg/m^2^ for females.^4^ MMI was defined as appendicular lean mass, measured in kg, divided by the squared standing height. Standing height was measured using a Seca 202 stadiometer measured in metre (m). Dual‐energy X‐ray absorptiometry (DXA), the gold standard for appendicular lean mass measurement, was only available in about 20,000 UKB participants. Thus, we used a conversion equation with high predictive ability (*R* ^2^ > 98%) to predict bioelectrical impedance analysis (BIA) appendicular lean mass using multivariable linear regression with appendicular fat‐free mass and sex as predictors.^18^ Appendicular fat‐free mass was derived by summing up left and right leg fat‐free mass and left and right arm fat‐free mass. Appendicular fat‐free mass components were measured using BIA with a Tanita BC418MA machine.

Severe sarcopenia was defined as confirmed sarcopenia with a slow gait speed, as an indicator of low physical performance.^4^ Self‐reported walking pace (categorised as slow, average or brisk) was used to proxy gait speed. A previous study, aiming to investigate the usefulness of self‐reported walking pace as a marker of physical performance, determined that it can serve as a reliable marker of measured walking speed.^19^ To proxy gait speed, the walking pace was dichotomized into slow or normal (average or brisk pace).

Overall, the population of the study was divided into four groups based on the sarcopenia categories: non‐sarcopenia, probable sarcopenia (without confirmed or severe sarcopenia), confirmed sarcopenia (without severe sarcopenia) and severe sarcopenia. Given the low number of UK Biobank participants with confirmed but not severe sarcopenia (*N* = 734) and severe sarcopenia (*N* = 200), these categories were analysed together. However, even when these categories were combined, the small number of cases did not allow for analyses by cancer type. For that reason, we also performed analyses after combining the populations of probable, confirmed and severe sarcopenia.

Sarcopenic obesity, which is defined as the coexistence of obesity and sarcopenia, is a condition of reduced lean body mass in the context of excess adiposity.^4^ Sarcopenic obesity analyses included participants with probable, confirmed and severe sarcopenia, who presented the additional characteristic of obesity (body mass index [BMI] ≥ 30 kg/m^2^).

### 2.3 Covariates

Through interviews and questionnaires, information was collected at baseline, primarily self‐reported, covering various aspects, including demographics (age, sex), socio‐economic (Townsend deprivation index [TDI]), lifestyle characteristics (smoking status and intensity, alcohol intake, metabolic equivalent of task [MET], sedentary behaviour, 9‐item dietary intake score), non‐steroidal anti‐inflammatory drugs use [NSAID], anthropometric measures (BMI), medical history of comorbidities (cardiovascular disease, diabetes, liver‐related non‐cancer illness or kidney failure, inflammatory bowel disease, chronic respiratory non‐cancer illness, heart failure), family history of cancer and sex‐specific factors (female: menopausal status, hormone replacement therapy [HRT], oral contraceptive use, age at menarche and mammographic history; male: testosterone and sex hormone binding globulin concentrations [SHBG]). More details on the definition of covariates are provided in Appendix S1.

### 2.4 Outcome definition

We obtained incident cancer information through linkage to the national cancer registries across England, Wales, and Scotland, up until June 2023. The International Classification of Diseases 9th (ICD‐9) and 10th (ICD‐10) revision codes were employed to define 19 specific cancer types. We defined malignant cancers with behaviour codes 3 (malignant, primary site) or 5 (malignant, microinvasive) and using ICD‐10/ICD‐9 codes as follows: all cancers (C00‐C97/140‐208), oral (C00‐C14/140‐149), oesophageal (C15/150), gastric (C16/151), colorectal (C18‐C20/153‐154), liver (C22/155), gallbladder (C23/156), pancreatic (C25/157), lung (C34/162), hematologic (C42, C81‐C96/200‐208), melanoma (C43/172), kidney (C64‐C65/189), bladder (C67/188), and brain (C71/191) for both sexes, breast (C50/174), cervical (C53/180), endometrial (C54/182), ovarian (C56/183) for females and prostate (C61/185) for males (Appendix S2). Breast cancer was further categorised as pre‐ and post‐menopausal according to age at cancer diagnosis (the cut‐off age was considered to be 52 years based on the mean age for menopause at baseline). Moreover, female participants with hysterectomy (*N* = 38,560) before recruitment were excluded from analyses of cervical/endometrial cancer, and female participants with bilateral oophorectomy (*N* = 15,531) before recruitment were excluded from analyses of ovarian cancer. As many participants have numerous cancer diagnoses, we identified the first primary incident cancer cases utilising the diagnosis date, ICD‐9/10, morphology and histology (Appendix S2). We considered incident cancer diagnoses recorded up to December 31, 2020 (Figure S1). We calculated person‐years from the recruitment date to the date of the first diagnosis of cancer, death, or the last date of follow‐up, whichever came first.

### 2.5 Study population

**FIGURE 1:**
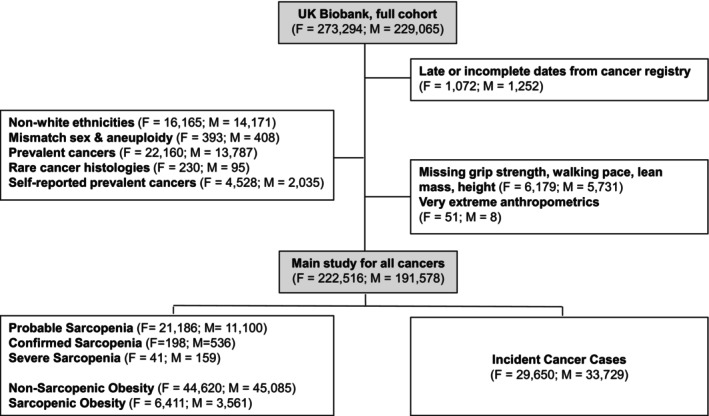
Flowchart of the study.

Ultimately, the final sample size was 414,094 participants, of which 63,379 had an incident cancer diagnosis (female = 29,650, male = 33,729) (Figure 1). The detailed exclusion criteria are described in Appendix S3.

### 2.6 Statistical analysis

Descriptive statistics are presented by sex, since the sarcopenia definition contains sex‐specific cut‐offs,^4^ and the sexual dimorphism of obesity has been demonstrated in the literature. For continuous variables, the mean and standard deviation (SD) or the median and interquartile range (IQR) are provided, depending on the normality of the distribution, which was assessed by the Anderson‐Darling normality test.^20^ Categorical variables are expressed as frequencies and percentages.

Cox proportional hazard models, maximising likelihood with Efron's approach,^21^ were used to estimate hazard ratios (HRs) and 95% confidence intervals (CIs) of sarcopenia stages and its components with total and site‐specific cancer incidence. We added interaction terms for sex and examined associations separately in women and men. The proportional hazard assumption was checked by tests based on Schoenfeld residuals showing no significant violations of the assumption.

We additionally examined linear associations of continuous grip strength and ΜΜΙ with cancer risk. To investigate potential nonlinear associations, we fitted restricted cubic splines with four knots at the 5th, 35th, 65th and 95th percentiles in Cox models, by sex.

We used two Cox regression models. Model 1 was adjusted for age (in years), sex (female, male), BMI (kg/m^2^), TDI (continuous), smoking status (never/former/current) and intensity, frequency of alcohol intake (never, 1–3 times/month, 1–2 times/week, >2 times/week), MET in minutes/week (as the sum of MET from moderate and vigorous physical activity and walking) and family history of cancers (merging history of father, mother and siblings; no/yes). In model 2, we further adjusted for a 9‐item dietary intake score based on previous literature (Appendix S1), sedentary behaviour (as the sum of hours spent watching television, using a computer, or driving), baseline presence of cardiovascular disease (no/yes), diabetes status (no/yes), NSAID use (no/yes) and for female‐specific cancers, we also adjusted for menopausal status (no/yes), ever oral contraceptive use (no/yes), hormone replacement therapy (no/yes) and age (years) at menarche. For prostate cancer, we further adjusted model 2 for testosterone and SHBG concentrations (continuously), and for breast cancer, we also adjusted for mammography history (no/yes). We re‐applied both models incorporating interaction terms between sex and sarcopenia components. Appendix S2 presents the percentages of missing values of the main study confounders, and the percentage was low for most variables. Participants with missing values were excluded from the analyses at the level of each statistical model and are not part of the main study exclusions presented in Figure 1 and Appedix S3 to maximise the sample size per investigated association. In the results section, descriptions will be based on model 2. For both sexes, cancer types with lower than 10 cases were ignored from analyses.

To minimise potential reverse causation, we conducted a sensitivity analysis after excluding 9230 (F = 4335, M = 4895) participants who had any type of cancer diagnosis or died within a period of 2 years after recruitment. Benjamini–Hochberg false discovery rate (FDR) analysis was implemented to adjust for multiple testing for the different cancers.^22^ Statistical significance was set at *p* < .05 in descriptive analyses. All analyses were performed using R version 4.3.1.

## 3 RESULTS

A total of 414,094 participants were included in this study, with a mean (SD) age of 56.4 (8.1) years and a median follow‐up of 11.7 years, during which 63,379 participants developed cancer. A total of 32,286 participants had probable sarcopenia and 934 participants had confirmed/severe sarcopenia at baseline (Table 1). Compared to individuals without sarcopenia, those with sarcopenia or probable sarcopenia were on average older, had lower physical activity, consumed less alcohol, were more likely to have cardiovascular disease, while women were more likely to have ever taken HRT and less likely to have conducted a mammography (Table 1). The same contrast of characteristics was evident when comparing participants with sarcopenic obesity to participants with obesity but without sarcopenia, except in female participants with sarcopenic obesity who were more likely to have conducted a mammography (Table 1). The number of incident cancer cases by sarcopenia category and by sarcopenia components are presented in Tables S1 and S2, respectively. Gallbladder and cervical cancer were not included in the analyses due to the low number of cases (below 10 cancer cases for each cancer overall). Results of the association with cancer incidence will be presented in the following order: combined probable‐confirmed‐severe sarcopenia, confirmed‐severe sarcopenia, probable sarcopenia, grip strength (categorical according to sarcopenia definition cut‐offs, continuous linear and non‐linear), muscle mass index (categorical according to definition cut‐offs, continuous linear and non‐linear), walking pace and sarcopenic obesity.

**TABLE 1:** Baseline characteristics by sarcopenia and sex.

Feature	Overall (F = 222,516; M = 191,578)	No sarcopenia (F = 201,091; M = 179,783)	Probable sarcopenia^a^ (F = 21,186; M = 11,100)	Confirmed and severe sarcopenia (F = 239; M = 695)	Non‐sarcopenic obesity (F = 44,620; M = 45,085)	Sarcopenic obesity (F = 6411; M = 3561)
Follow up (years)						
F	11.75 (10.9, 12.5)	11.8 (10.9, 12.5)	11.5 (10.7, 12.3)	11.3 (10.6, 12.2)	11.8 (10.8, 12.5)	11.6 (10.7, 12.4)
M	11.63 (10.7, 12.5)	11.7 (10.7, 12.5)	11.3 (10.4, 12.2)	11.2 (8.5, 12.2)	11.6 (10.7, 12.5)	11.2 (9.8, 12.2)
Age (years)						
F	56.2 (7.98)	55.79 (7.99)	60.02 (6.82)	61.02 (6.15)	56.29 (7.8)	59.7 (6.81)
M	56.57 (8.12)	56.36 (8.13)	59.66 (7.48)	61.33 (6.81)	56.51 (7.91)	59.74 (7.25)
BMI (kg/m^2^)						
F	26.04 (23.4, 29.6)	25.9 (23.3, 29.5)	27.1 (24.2, 31.1)	19.1 (17.9, 20.1)	33.1 (31.3, 36.1)	33.6 (31.5, 36.8)
M	27.31 (25, 30.1)	27.3 (25, 30)	27.9 (25.4, 31.1)	21 (19.5, 22.2)	32.3 (31, 34.6)	32.9 (31.2, 35.5)
METs (min/week)						
F	1177.5 (132, 2817)	1215 (179, 2844)	796 (0, 2346)	792 (0, 2606.2)	777 (0, 2175)	462 (0, 1659.5)
M	1426 (384, 3279)	1464 (398, 3324)	933 (66, 2533)	933 (0, 2659.5)	1125 (198, 2910)	688 (0, 1998)
Alcohol frequency intake						
Never drinker						
F	17,345 (7.8)	14,524 (7.2)	2791 (13.2)	30 (12.6)	4427 (9.9)	1116 (17.4)
M	9303 (4.9)	8188 (4.6)	1034 (9.3)	81 (11.7)	2385 (5.3)	372 (10.5)
1–3 times/month						
F	60,664 (27.3)	53,713 (26.7)	6883 (32.5)	68 (28.5)	16,412 (36.8)	2603 (40.6)
M	29,591 (15.5)	27,313 (15.2)	2151 (19.4)	127 (18.3)	8439 (18.7)	822 (23.2)
1–2 times/week						
F	59,203 (26.6)	53,886 (26.8)	5261 (24.9)	56 (23.4)	11,658 (26.1)	1439 (22.5)
M	50,423 (26.3)	47,381 (26.4)	2909 (26.3)	133 (19.2)	12,908 (28.6)	1003 (28.3)
>2 times/week						
F	85,171 (38.3)	78,852 (39.2)	6234 (29.4)	85 (35.6)	12,091 (27.1)	1247 (19.5)
M	102,124 (53.3)	96,790 (53.9)	4982 (45)	352 (50.8)	21,324 (47.3)	1351 (38.1)
Smoking status						
Never smoker						
F	131,163 (59.1)	118,848 (59.3)	12,174 (57.8)	141 (59)	25,552 (57.5)	3568 (56)
M	93,547 (49)	88,175 (49.2)	5079 (46)	293 (42.2)	19,377 (43.2)	1426 (40.4)
Former smoker						
F	70,939 (32)	63,939 (31.9)	6946 (33)	54 (22.6)	15,189 (34.2)	2253 (35.4)
M	73,903 (38.7)	69,184 (38.6)	4522 (41)	197 (28.4)	20,495 (45.7)	1715 (48.6)
Current smoker						
F	19,696 (8.9)	17,703 (8.8)	1949 (9.3)	44 (18.4)	3716 (8.4)	550 (8.6)
M	23,490 (12.3)	21,856 (12.2)	1430 (13)	204 (29.4)	5013 (11.2)	389 (11)
Smoking intensity (pack years)						
F	0 (0, 16.5)	0 (0, 16.5)	0 (0, 16.5)	0 (0, 16.5)	0 (0, 17)	0 (0, 17)
M	0 (0, 16.5)	0 (0, 16.5)	0 (5, 19)	0 (6, 24.8)	0 (0, 23)	0 (0, 25.12)
TDI						
F	−2.27 (−3.7, 0.2)	−2.3 (−3.7, 0.1)	−1.9 (−3.5, 1)	−1.9 (−3.3, 0.5)	−1.8 (−3.4, 1)	−1.1 (−3.1, 2)
M	−2.26 (−3.7, 0.3)	−2.3 (−3.7, 0.2)	−1.3 (−3.2, 2)	−0.5 (−2.7, 3.4)	−2 (−3.6, 0.8)	−0.6 (−2.9, 2.6)
Family history of cancer (yes)						
F	79,258 (38.8)	71,115 (38.6)	8064 (41)	79 (37.3)	16,229 (39.3)	2517 (41.9)
M	66,942 (39.3)	62,674 (39.2)	4021 (40.7)	247 (40.4)	15,997 (39.4)	1319 (41.3)
Dietary intake score^b^						
F	4 (3, 5)	4 (3, 5)	4 (3, 5)	4 (3, 5.5)	4 (4, 5)	5 (4, 5)
M	5 (4, 6)	5 (4, 6)	5 (4, 6)	5 (4, 6)	5 (4, 6)	5 (4, 6)
Sedentary behaviour (h)						
F	4 (3, 5.5)	4 (3, 5.5)	4.5 (3, 6)	4 (2.8, 5)	4.5 (3.5, 6)	5 (3.5, 6)
M	5 (3.5, 6.5)	5 (3.5, 6.5)	5 (3.5, 7)	4.5 (3, 6)	5.5 (4, 7.5)	6 (4, 7.5)
Diabetes (yes)						
F	7395 (3.3)	6073 (3)	1317 (6.2)	5 (2.1)	3440 (7.7)	830 (12.9)
M	12,118 (6.3)	10,528 (5.9)	1559 (14)	31 (4.5)	5571 (12.4)	879 (24.7)
Cardiovascular disease (yes)						
F	17,551 (7.9)	14,332 (7.1)	3183 (15)	36 (15.1)	4745 (10.6)	1306 (20.4)
M	32,071 (16.7)	28,937 (16.1)	2979 (26.8)	155 (22.3)	9827 (21.8)	1199 (33.7)
NSAID use (yes)						
F	58,701 (26.5)	52,090 (26.1)	6548 (31)	63 (26.6)	14,452 (32.5)	2372 (37.1)
M	55,884 (29.4)	51,502 (28.8)	4187 (38)	195 (28.3)	16,709 (37.3)	1682 (47.6)
Prior chronic disease^c^ (yes)						
F	9367 (4.2)	7898 (3.9)	1434 (6.8)	35 (14.6)	1971 (4.4)	498 (7.8)
M	9579 (5)	8556 (4.8)	922 (8.3)	101 (15.3)	2332 (5.2)	339 (9.5)
Sex specific adjustments, male						
SHBG (nmol/L)						
M	36.98 (28, 48)	36.9 (28, 47.9)	37.7 (28.2, 49.5)	54.4 (44.3, 67.9)	31.8 (24, 41.3)	33.1 (24.7, 42.9)
Testosterone (nmol/L)						
M	11.67 (9.5, 14.2)	11.7 (9.5, 14.2)	11.1 (9, 13.7)	13.3 (10.8, 16.3)	10.3 (8.4, 12.5)	9.8 (7.8, 12.1)
Sex specific adjustments, female						
Menopause (yes)						
F	127,359 (70.5)	11,3024 (68.8)	14,174 (87.9)	161 (95.3)	25,987 (71.9)	4333 (87.6)
Age at menarche (years)						
F	13 (12, 14)	13 (12, 14)	13 (12, 14)	14 (12, 15)	13 (11, 14)	12 (11, 14)
HRT use (yes)						
F	80,271 (37.7)	69,686 (36.1)	10,487 (53.2)	98 (48)	17,082 (38.4)	3380 (52.9)
Mammography history (yes)						
F	44,944 (21.1)	43,103 (22.3)	1824 (9.2)	17 (8.3)	35,375 (79.4)	5772 (90.2)
Sarcopenia components						
Grip strength (kg)						
F	23.5 (19.5, 28)	24 (21, 28)	13 (11, 14.5)	13 (10, 14.8)	24 (20.5, 28)	13 (10.5, 14.5)
M	40 (34, 45.5)	40 (35, 46)	23 (20, 25)	23 (20, 25)	40.5 (35, 46.5)	23 (20, 25)
ALM (kg)						
F	18.09 (16.7, 19.6)	18.1 (16.8, 19.7)	17.6 (16.3, 19.3)	14.2 (13.5, 14.9)	20.8 (19.3, 22.4)	20.1 (18.7, 21.8)
M	26.73 (24.4, 29.3)	26.8 (24.5, 29.3)	25.5 (23.3, 28.4)	19.5 (18.3, 20.9)	30.5 (28.4, 33)	29.1 (26.9, 31.6)
AFM (kg)						
F	19.2 (17.8, 20.8)	19.2 (17.9, 20.9)	18.7 (17.3, 20.5)	15.1 (14.4, 15.8)	22 (20.5, 23.7)	21.3 (19.8, 23.1)
M	28.4 (26, 31.1)	28.5 (26.1, 31.1)	27.1 (24.8, 30.1)	20.9 (19.6, 22.3)	32.3 (30.1, 34.9)	30.9 (28.6, 33.5)
MMI (kg)						
F	6.81 (6.4, 7.4)	6.8 (6.4, 7.3)	6.9 (6.4, 7.5)	5.4 (5.2, 5.4)	7.9 (7.5, 8.4)	7.9 (7.5, 8.5)
M	8.62 (8, 9.3)	8.6 (8, 9.3)	8.6 (7.9, 9.4)	6.7 (6.4, 6.9)	9.8 (9.4, 10.4)	9.8 (9.3, 10.5)
Walking pace						
Slow						
F	16,127 (7.2)	11,632 (5.8)	4454 (21)	41 (17.2)	6772 (15.2)	2439 (38)
M	13,183 (6.9)	10,520 (5.9)	2504 (22.6)	159 (22.9)	5340 (11.8)	1254 (35.2)
Average						
F	117,411 (52.8)	10,5495 (52.5)	11,802 (55.7)	114 (47.7)	29,286 (65.6)	3405 (53.1)
M	99,550 (52)	93,432 (52)	5801 (52.3)	317 (45.6)	27,822 (61.7)	1830 (51.4)
Brisk						
F	88,978 (40)	83,964 (41.8)	49,30 (23.3)	84 (35.1)	8562 (19.2)	567 (8.8)
M	78,845 (41.2)	75,831 (42.2)	2795 (25.2)	219 (31.5)	11,923 (26.4)	477 (13.4)

*Note*: Data are presented as absolute number (%), median (25th, 75th percentile) or mean ± standard deviation (SD).

Abbreviations: AFM, appendicular fat mass; ALP, appendicular lean mass; BMI, body mass index; MET, metabolic equivalent of task; MMI, muscle mass index; NSAID: non‐steroidal anti‐inflammatory drugs; TDI, Townsend deprivation index; M, male; F, female.

^a^ Without confirmed and severe sarcopenia.

^b^ Dietary Intake was derived as described in Appendix S1.

^c^ History of comorbidities (cardiovascular disease, diabetes, liver‐related non‐cancer illness or kidney failure, inflammatory bowel disease (non‐cancer illness)).

### 3.1 Associations with sarcopenia categories

**FIGURE 2:**
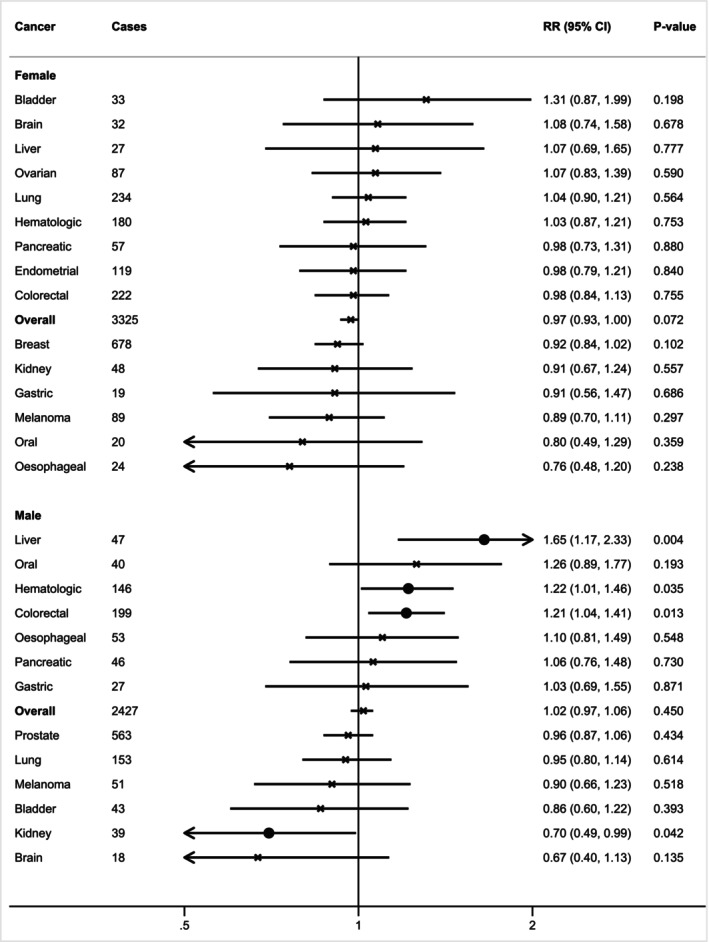
Sex‐specific associations between probable/confirmed/severe sarcopenia versus non‐sarcopenia and cancer incidence for 19 cancers. The same associations are observed for low grip strength, a main component of sarcopenia (<27 kg for males and <16 kg for females), which is included in the definition of all the sarcopenia categories. Data are presented in hazard ratio with 95% confidence intervals. X indicates statistically non‐significant associations; • indicates statistically significant associations; * indicates statistically significant associations after adjustment for false discovery rate (FDR). Probable sarcopenia was defined as low grip strength (<27 kg for males and <16 kg for females). Confirmed sarcopenia was defined as low grip strength and low muscle mass index (<7 kg/m^2^ for males and <5.5 kg/m^2^ for females). Severe sarcopenia was defined as low grip strength, low muscle mass index, and slow walking pace. Models adjusted for age, gender (female, male), body mass index (BMI, continuous), Townsend deprivation index (TDI, continuous), smoking status (never/former/current), alcohol frequency intake (never drinker, 1–3 times/month, 1–2 times/week, >3 times/week), metabolic equivalent of task (MET‐minutes/week, continuous), family history of (no/yes), 9‐item dietary intake score (continuous), sedentary behaviour (continuous), cardiovascular disease (no/yes), diabetes (no/yes), nonsteroidal anti‐inflammatory drugs (NSAID) use (no/yes). Prostate cancer was further adjusted for testosterone (continuous) and sex hormone‐binding globulin (SHBG, continuous) concentrations. Breast cancer was further adjusted for history of mammography (no/yes), while additional adjustments for female‐specific cancers were menopausal status (no/yes), oral contraceptive use (no/yes), hormone replacement therapy (HRT) use (no/yes), and age at menarche (continuous).

Nominal statistical significant associations were observed for combined probable, confirmed, and severe sarcopenia with a higher risk of liver (HR: 1.65, 95% CI: 1.17–2.33), haematological (HR: 1.22, 95% CI: 1.01–1.46) and colorectal cancer (HR: 1.21, 95% CI: 1.04–1.41) in males (Figure 2, Table S3). In females, the latter associations were null (*p*_interaction by sex_ = .12, .173, .044, respectively). Combined probable, confirmed, and severe sarcopenia was associated with a lower risk of kidney cancer in males (HR: 0.7, 95% CI: 0.49–0.99), but not in females (*p*_interaction_ = .261). Combined probable, confirmed, and severe sarcopenia was also associated with a lower risk of breast cancer in post‐menopausal women (HR: 0.92, 95% CI: 0.84–1.00). None of the associations achieved FDR significance. In the sensitivity analysis, after excluding 9230 participants who experienced any cancer type or death within the first 2 years of follow‐up, the associations of probable, confirmed, and severe sarcopenia with a higher risk for liver and colorectal cancer as well as a lower risk for kidney cancer in males remained statistically significant (Table S4).

The limited number of participants with confirmed (*N* = 734) and severe sarcopenia (*N* = 200) did not allow for analyses by cancer type, even when pooled together (Table S1). Confirmed and severe sarcopenia were not associated with risk for overall cancer in neither females (HR: 1.06, 95% CI: 0.77–1.46) nor males (HR: 0.98, 95% CI: 0.82–1.17).

For probable sarcopenia, the significant associations for liver (HR: 1.68, 95% CI: 1.19–2.39, FDR‐significant), haematological (HR: 1.21, 95% CI: 1–1.46), and colorectal cancer (HR: 1.26, 95% CI: 1.08–1.47, FDR‐significant) in males were observed (Table S5), but not in females (*p*_interaction_ = .086, .223, .016, respectively), and significant associations were not observed for kidney and post‐menopausal breast cancer. In the sensitivity analysis, the associations with liver and colorectal cancer remained statistically significant in men (Table S6).

### 3.2 Associations with sarcopenia components

#### 3.2.1 Grip strength

Low grip strength, as an individual dichotomous component of sarcopenia, exhibited the same associations as mentioned above for the combined population of probable, confirmed, and severe sarcopenia (Figure 2, Tables S3 and S4), which is expected since grip strength is the common variable included in all of these definitions.

When considering grip strength as a continuous variable, the estimated linear and non‐linear associations with cancer risk in males and females are presented in Table S7 and Figure S3, respectively. Higher grip strength by 1 SD was linearly associated with lower risk for any type of cancer (HR: 0.99, 95% CI: 0.97–1.0) and liver cancer (HR: 0.82, 95% CI: 0.73–0.93, FDR‐significant) in males, with higher risk for melanoma in males (HR: 1.08, 95% CI: 1–1.16), and higher risk for any type of cancer (HR: 1.04, 95% CI: 1.03–1.05, FDR‐significant) in females, as well as higher risk of breast cancer in post‐menopausal women (HR: 1.03, 95% CI: 1.01–1.06) (Table S7). Some evidence for non‐linear associations was observed for oral and haematological malignancies in males, as well as endometrial cancer, but data in the tails of the distributions were sparse (Figure S3).

#### 3.2.2 Muscle mass index

**FIGURE 3:**
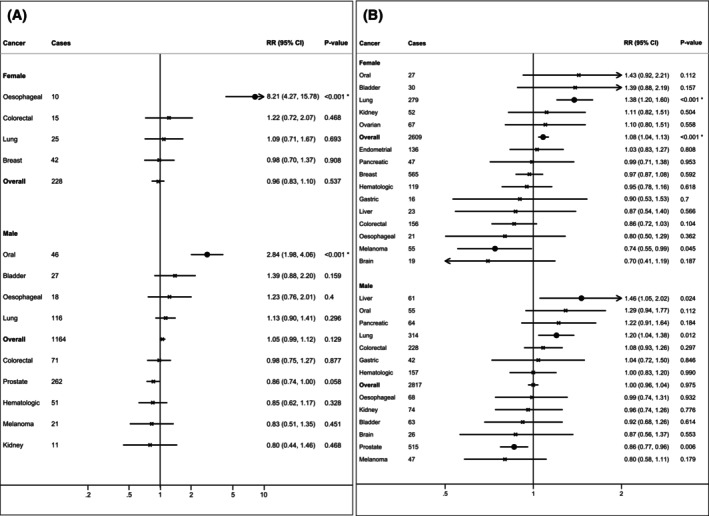
Sex specific associations for the other components of sarcopenia, including low muscle mass index and slow walking pace. (A) Associations between low muscle mass index (<7 kg/m^2^ for males and <5.5 kg/m^2^ for females) versus normal muscle mass index and cancer incidence for 19 cancers. (B) Associations between slow walking pace versus normal walking pace and cancer incidence for 19 cancers. Data are presented in hazard ratio with 95% confidence intervals. X indicates statistically non‐significant associations; • indicates statistically significant associations; * indicates statistically significant associations after adjustment for false discovery rate (FDR). Models adjusted for age, gender (female, male), body mass index (BMI, continuous), Townsend deprivation index (TDI, continuous), smoking status (never/ former/current), alcohol frequency intake (never drinker, 1–3 times/month, 1–2 times/week, >3 times/week), metabolic equivalent of task (MET‐minutes/week, continuous), family history of (no/yes), 9‐item dietary intake score (continuous), sedentary behaviour (continuous), cardiovascular disease (no/yes), diabetes (no/yes), nonsteroidal anti‐inflammatory drugs (NSAID) use (no/yes). Prostate cancer was further adjusted for testosterone (continuous) and sex hormone‐binding globulin (SHBG, continuous) concentrations. Breast cancer was further adjusted for history of mammography (no/yes), while additional adjustments for female‐specific cancers were menopausal status (no/yes), oral contraceptive use (no/yes), hormone replacement therapy (HRT) use (no/yes), and age at menarche (continuous).

Low MMI, as an individual dichotomous component of sarcopenia, was FDR‐significantly associated with a higher risk of oesophageal cancer in females (HR: 8.21, 95% CI: 4.27–15.78) but not in males (*p*_interaction_ < .001), and a higher risk of oral cancer in males (HR: 2.84, 95% CI: 1.98–4.06) (Figure 3A, Table S8). In the sensitivity analysis, the association of low MMI with a higher risk for oesophageal cancer in females and oral cancer in males remained statistically significant (Table S9).

When considering MMI as a continuous variable, the estimated linear and non‐linear associations with cancer risk are presented in Table S10 and Figure S4, respectively. Higher MMI by 1 SD was FDR‐significantly linearly associated with lower risk for oesophageal (HR: 0.56, 95% CI: 0.44–0.72), liver (HR: 0.61, 95% CI: 0.46–0.81), and lung cancer (HR: 0.77, 95% CI: 0.69–0.86) in females, as well as lower risk for lung cancer in males (HR: 0.79, 95% CI: 0.72–0.87), and was also linearly associated with higher risk for haematological malignancies (HR: 1.22, 95% CI: 1.11–1.36), melanoma (HR: 1.2, 95% CI: 1.05–1.37), and any type of cancer (HR: 1.04, 95% CI: 1.01–1.06) in females, as well as higher risk for haematological (HR: 1.23, 95% CI: 1.12–1.35), melanoma (HR: 1.28, 95% CI: 1.13–1.44) and prostate cancer (HR: 1.13, 95% CI: 1.06–1.21) in males (Table S10). Some evidence for non‐linear associations was observed for oral, melanoma, and prostate cancer in males, as well as oesophageal, breast, and overall cancer in females, but data in the tails of the distributions were sparse (Figure S4).

#### 3.2.3 Walking pace

Slow walking pace, as an indicator of low physical performance and an individual component of sarcopenia, was FDR‐significantly associated with a higher risk of any type of cancer (HR: 1.08, 95% CI: 1.04–1.13) in females but not in males (*p*_interaction_ = .008) (Figure 3B, Table S11). Slow walking pace was associated with a higher risk of lung cancer in males (HR: 1.2, 95% CI: 1.04–1.38) and was FDR‐significantly associated with a higher risk of lung cancer in females (HR: 1.38, 95% CI: 1.2–1.6) (*p*_interaction_ = .147). Slow walking pace was associated with a higher risk of liver cancer (HR: 1.46, 95% CI: 1.05–2.02) in males, but not in females (*p*_interaction_ = .066) (Figure 3B, Table S11). Slow walking pace was not associated with the risk of oral cancer in the sex‐specific analyses but was associated with a higher risk for oral cancer in the overall population (HR: 1.33, 95% CI: 1.02–1.73). Moreover, slow walking pace was associated with a higher risk of melanoma in females (HR: 0.74, 95% CI: 0.55–0.99) but not in males (*p*_interaction_ = .709). Finally, it was associated with a lower risk of prostate cancer in males (HR: 0.86, 95% CI: 0.77–0.96) (Figure 3B, Table S11). In the sensitivity analyses, the associations that remained significant consisted of liver, lung and prostate cancer in males, as well as any type of cancer and lung cancer in females (Table S12).

#### 3.2.4 Sarcopenic obesity

**FIGURE 4:**
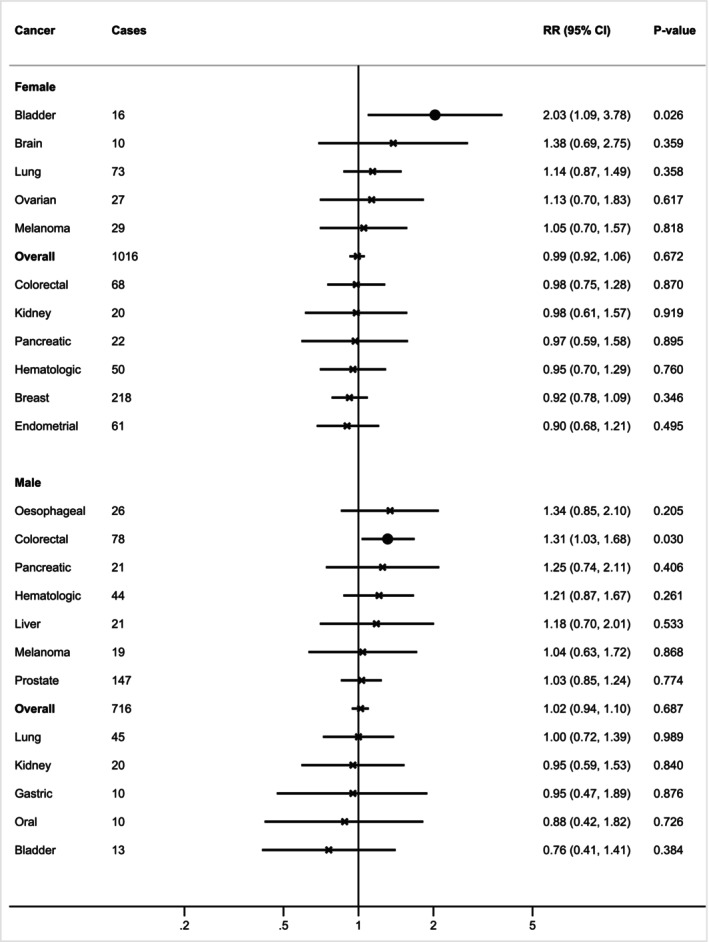
Sex‐specific associations between sarcopenic obesity versus non‐sarcopenic obesity and cancer incidence for 19 cancers. Data are presented in hazard ratio with 95% confidence intervals. X indicates statistically non‐significant associations; • indicates statistically significant associations; * indicates statistically significant associations after adjustment for false discovery rate (FDR). Models adjusted for age, gender (female, male), body mass index (BMI, continuous), Townsend deprivation index (TDI, continuous), smoking status (never/ former/current), alcohol frequency intake (never drinker, 1–3 times/month, 1–2 times/week, >3 times/week), metabolic equivalent of task (MET‐minutes/week, continuous), family history of (no/yes), 9‐item dietary intake score (continuous), sedentary behaviour (continuous), cardiovascular disease (no/yes), diabetes (no/yes), nonsteroidal anti‐inflammatory drugs (NSAID) use (no/yes). Prostate cancer was further adjusted for testosterone (continuous) and sex hormone‐binding globulin (SHBG, continuous) concentrations. Breast cancer was further adjusted for history of mammography (no/yes), while additional adjustments for female‐specific cancers were menopausal status (no/yes), oral contraceptive use (no/yes), hormone replacement therapy (HRT) use (no/yes), and age at menarche (continuous).

Among individuals living with obesity, sarcopenic obesity when compared to non‐sarcopenic obesity was associated with a higher risk of colorectal cancer in males (HR: 1.31, 95% CI: 1.03–1.68) but not in females (*p*_interaction_ = .111), and with a higher risk of bladder cancer in females (HR: 2.03, 95% CI: 1.09–3.78) but not in males (*p*_interaction_ = .027) (Figure 4, Table S13). None of the associations achieved FDR significance. In the sensitivity analysis, only the association of sarcopenic obesity with a higher risk of colorectal cancer in males remained statistically significant (Table S14).

### 3.3 Summary of associations

**FIGURE 5:**
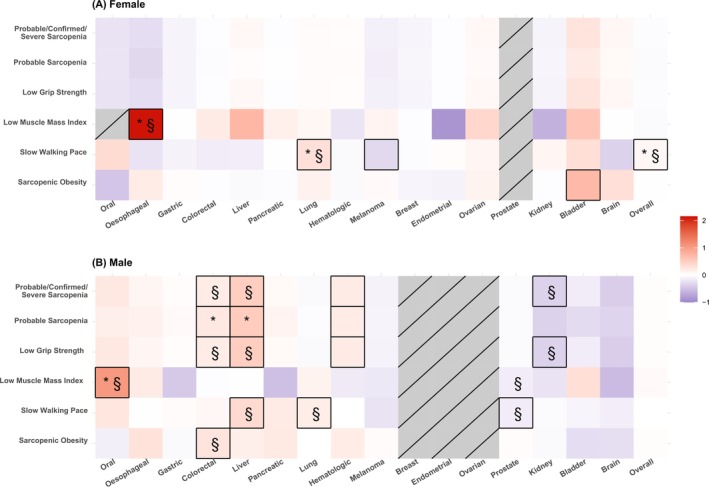
Heatmap for the associations of sarcopenia, sarcopenia components and sarcopenic obesity with the incidence of 17 different types of cancer in UK Biobank in (A) females and (B) males. Colour represents the direction of the log hazard ratio (red: higher risk for cancer; blue: lower risk for cancer) from the fully adjusted model. The colour depth indicates association magnitudes (the darker the stronger). The dashed squares indicate non‐applicable associations or associations with zero cases. The boxed full‐line squares denote nominally statistically significant associations. The symbol (*) denotes significance after correction for multiple testing (FDR < 5%). The symbol (§) denotes the associations that survived the sensitivity analysis of excluding participants who had any type of cancer diagnosis or died within a period of 2 years after recruitment. Models adjusted for age, gender (female, male), body mass index (BMI, continuous), Townsend deprivation index (TDI, continuous), smoking status (never/former/current), alcohol frequency intake (never drinker, 1–3 times/month, 1–2 times/week, >3 times/week), metabolic equivalent of task (MET‐minutes/week, continuous), family history of (no/yes), 9‐item dietary intake score (continuous), sedentary behaviour (continuous), cardiovascular disease (no/yes), diabetes (no/yes), nonsteroidal anti‐inflammatory drugs (NSAID) use (no/yes). Prostate cancer was further adjusted for testosterone (continuous) and sex hormone‐binding globulin (SHBG, continuous) concentrations. Breast cancer was further adjusted for history of mammography (no/yes), while additional adjustments for female‐specific cancers were menopausal status (no/yes), oral contraceptive use (no/yes), hormone replacement therapy (HRT) use (no/yes), and age at menarche (continuous).

Figure 5 summarises our findings by sarcopenia category and by sex for every studied cancer type, highlighting associations that survived the FDR multiple testing correction and the sensitivity analysis of excluding participants who had any type of cancer diagnosis or died within a period of 2 years after recruitment.

## 4 DISCUSSION

Our study utilised prospective data from the UK Biobank to examine the association of sarcopenia and its components with the risk of 19 cancers. Combined probable, confirmed, and severe sarcopenia was associated with a higher risk of liver and colorectal malignancies in males, but not in females. Furthermore, associations were observed between the individual components of sarcopenia and several cancers, mainly of the gastrointestinal tract. Finally, compared to non‐sarcopenic obesity, sarcopenic obesity was also associated with a higher risk of colorectal cancer in males.

The potential mechanistic link between sarcopenia and cancer development is yet to be clarified. A proposed hypothesis suggests that elevated inflammatory cytokines that accompany sarcopenia could be responsible for inflammation‐induced DNA damage, consequently increasing the risk of carcinogenesis.^14^, ^23^ We found that probable, confirmed, and severe sarcopenia is associated with a higher risk of liver and colorectal malignancies in males. It has been reported that healthy development and composition of lean muscle mass contribute to glucose homeostasis maintenance,^24^, ^25^ which in turn has been linked to a lower cancer risk, including colorectal and liver cancer.^26^, ^27^ Moreover, increased muscle mass improves metabolism and insulin sensitivity, as well as reduces adipose tissue deposition, a major risk factor for several cancers.^28^, ^29^, ^30^ Finally, there is a tight balance between skeletal muscle and immune system function and responsiveness, which, if disturbed, could mechanistically lead to carcinogenesis.^31^, ^32^

Grip strength has been proposed as a practical and inexpensive way to measure skeletal muscle strength.^4^ Low grip strength has been studied as a powerful predictor of poor patient outcomes, such as functional limitations, poor health‐related quality of life, increased hospitalisation and mortality.^4^ The reliable measurement and formulation of grip strength is also of essence for studies of sarcopenia. In a previous UKB study published in 2022, following comparisons with numerous different expressions of grip strength, such as absolute and relative to height, weight, body fat mass, and BMI, the authors concluded that the expression in its most basic form (kilogram) is sufficient for predicting cancer outcomes.^33^ The authors of this study found absolute grip strength to be inversely and linearly associated with risk of endometrial, gallbladder, kidney, breast, and overall cancer, but did not perform analyses by sex.^33^ Discrepancies between these associations and our results could be attributed to the newer version of the UK biobank database used in our analyses, as well as to the rigorous exclusions and adjustments that we followed. Based on our findings, lower grip strength was linearly associated with higher risk for overall cancer and liver cancer only in male participants, further adding to the importance of sex‐specific analyses. Moreover, low grip strength, as an individual dichotomous component of sarcopenia, was associated with higher risk of liver and colorectal cancer in males.

Although MMI is not utilised alone for the definition of sarcopenia categories in the 2019 EWGSOP definition, according to the 2010 EWGSOP definition, low MMI forms pre‐sarcopenia, a conceptual stage of sarcopenia.^5^ In our study, low MMI, based on the cut‐offs from the sarcopenia definition, was FDR‐significantly associated with a higher risk of oral cancer in males and oesophageal cancer in females. We were careful to adjust for smoking status and intensity, since low muscle mass has been correlated with heavier smoking and reflects an enhanced susceptibility to smoking‐related carcinogenesis.^34^ In a 2021 UKB study, fat‐free mass, which primarily refers to muscle mass even though it is not completely identical by definition, was reported to be a stronger predictor of overall cancer risk compared to fat mass in mutually adjusted models including an additional adjustment for height.^35^ In this study, fat‐mass to fat‐free mass ratio was associated with a higher risk of colon, oesophageal, and kidney cancers in men, as well as postmenopausal breast and endometrial cancers in women.^35^

Finally, we found that slow walking pace, as a marker of physical capability, was associated with a higher risk of lung and overall cancer in females, as well as liver and lung cancer in males. While our study is the first that demonstrates an association between walking speed and cancer incidence, walking pace has been associated with higher mortality among cancer survivors.^36^ Moreover, it has been suggested that gait speed can predict adverse outcomes related to sarcopenia, such as cognitive impairment, disability, falls, need for institutionalisation, and mortality.^37^ Nonetheless, the association with a higher risk for overall cancer establishes the foundation for future research and replication.

Sarcopenic obesity has been independently associated with higher mortality and increased treatment complications in cancer patients.^38^ In our study, sarcopenic obesity compared to non‐sarcopenic obesity was associated with a higher risk of colorectal cancer in males. This finding is of special interest, since obesity has served as a major well‐established risk factor for cancer. Our results highlight the potential addition of sarcopenia as a novel complementary characteristic of obesity for cancer prevention. Increased alertness towards the detection of sarcopenia in populations living with obesity is required to achieve timely referral for intervention and control of the repercussions of the condition.

Special focus should be directed to the sexual dimorphism of our results. Heterogeneity according to sex was observed for most of the associations in our study, including sarcopenia, grip strength, muscle mass index, walking pace and sarcopenic obesity. Especially for sarcopenia and sarcopenic obesity, associations with higher risk for cancer, namely liver and colorectal, were shown only in males. Sexual dimorphism has been a recurring finding in the research of anthropometric indices. The genetic basis for sex differences in obesity and lipid metabolism has been described.^39^ A large‐scale genome‐wide interaction study identified 44 loci with sex‐specific effects contributing to body shape definition.^40^ Men have significantly more skeletal muscle mass in comparison to women in both absolute terms and relative to body mass, with greater sex differences being observed in the upper compared to the lower body.^41^ Sexual dimorphism in strength has been attributed to greater male muscle mass and type II fibre areas, while differences are more pronounced in upper‐body than lower‐body muscles and in concentric than eccentric contractions.^42^ Furthermore, sex differences regarding the regulatory role of skeletal muscle have been discussed, and the higher insulin sensitivity of the female muscle has been linked to sex‐specific regulation of molecular metabolism.^43^

Results from our study propose that sarcopenia and the sarcopenia components should not be neglected as a potential risk factor for a variety of cancers in both sexes, but primarily in males. This knowledge, combined with the fact that sarcopenia is detectable and treatable, could signal new avenues in the clinical practice towards cancer prevention. Resistance training is currently the main treatment option to improve lean mass, strength, and physical function.^44^ Protein‐rich diet and protein supplementation could additionally benefit older adults with sarcopenia.^44^

The present study has several strengths. This was a prospective cohort study, with a well‐characterised large sample size of white middle‐aged and older adults, and long‐term follow‐up. The wide range of measured cancer risk factors allowed for rigorous adjustments. However, potential limitations should also be addressed. Firstly, although the UKB constitutes a large population‐based resource, generalisation of results requires caution since it is not representative of the general population in terms of deprivation and lifestyle. Secondly, body composition was measured using BIA, which is not considered the gold standard for the assessment of muscle mass, although the EWGSOP (2019) supports the use of BIA in the research setting for sarcopenia confirmation.^4^ Moreover, the combination of probable, confirmed, and severe sarcopenia was necessary due to the small number of confirmed and severe sarcopenia cases, which did not allow separate analyses, and thus these results are mainly driven from probable sarcopenia, which is characterised by low grip strength. In addition, the small number of sarcopenia cases did not allow for stratification of analyses by age. Finally, the use of a single measurement of body composition at baseline does not facilitate monitoring of alterations over time and their potential linkage to the outcomes of interest. Unfortunately, longitudinal measurements were available only for a very limited part of the population to allow for meaningful analyses.

## 5 CONCLUSIONS

Sarcopenia and its components were mainly associated with a higher incidence of gastrointestinal tract cancers in males. The combination of probable, confirmed, and severe sarcopenia, which was mainly driven by low grip strength in this study, was associated with a higher risk of colorectal and liver cancer in males. Low muscle mass index (MMI), as defined in the sarcopenia definition and also known as “pre‐sarcopenia”, was associated with a higher risk of oesophageal cancer in females and oral cancer in males. Finally, among male individuals living with obesity, sarcopenia as an individual clinical entity was shown to be associated with a higher risk of colorectal cancer. Moreover, the existence of sexual dimorphism in the associations of sarcopenia and sarcopenia components is highlighted as a point of reference for future studies. Increasing awareness regarding sarcopenia detection, as well as establishing frameworks to treat it, can hold benefits for people with sarcopenia and its components, including a potential reduction of cancer risk.

## AUTHOR CONTRIBUTIONS

**Panagiotis Filis:** Conceptualization; methodology; data curation; investigation; validation; formal analysis; visualization; writing – original draft; writing – review and editing. **Christos K. Papagiannopoulos:** Methodology; software; data curation; validation; formal analysis; writing – original draft; writing – review and editing; resources. **Georgios Markozannes:** Methodology; software; data curation; investigation; validation; formal analysis; writing – review and editing; writing – original draft; resources. **Christos V. Chalitsios:** Writing – review and editing; methodology; software; data curation; investigation. **Ioannis Zerdes:** Writing – review and editing; data curation; investigation. **Antonios Valachis:** Data curation; investigation; writing – review and editing. **Christopher Papandreou:** Data curation; investigation; writing – review and editing; resources. **Sofia Christakoudi:** Methodology; investigation; validation; writing – review and editing; supervision. **Konstantinos K. Tsilidis:** Conceptualization; methodology; project administration; visualization; writing – review and editing; writing – original draft; supervision; resources.

## CONFLICT OF INTEREST STATEMENT

All authors declare that they have no conflict of interest.

## ETHICS STATEMENT

UK Biobank was approved by the Northwest Multi‐Centre Research Ethics Committee (Ref: 11/NW/0382).

## Supporting information


**APPENDIX S1.** Supporting information.

## Data Availability

This work uses data provided by patients and collected by the NHS as part of their care and support. This work has been conducted using the UK Biobank resource under the application number 79696. The UK Biobank is an open access resource, and bona fide researchers can apply to use the UK Biobank dataset by registering and applying at http://ukbiobank.ac.uk/register-apply/. Further information is available from the corresponding author upon request. The source code is publicly available on GitHub (https://github.com/Papagiannopoulos/Sarcopenia).

## References

[1] Anker SD , Morley JE , von Haehling S . Welcome to the ICD‐10 code for sarcopenia. J Cachexia Sarcopenia Muscle. 2016;7(5):512‐514. 27891296 10.1002/jcsm.12147 PMC5114626

[2] Walston JD . Sarcopenia in older adults. Curr Opin Rheumatol. 2012;24(6):623‐627. 22955023 10.1097/BOR.0b013e328358d59b PMC4066461

[3] Rosenberg IH . Summary comments. Am J Clin Nutr. 1989;50(5):1231‐1233.

[4] Cruz‐Jentoft AJ , Bahat G , Bauer J , et al. Sarcopenia: revised European consensus on definition and diagnosis. Age Ageing. 2019;48(1):16‐31. 30312372 10.1093/ageing/afy169 PMC6322506

[5] Cruz‐Jentoft AJ , Baeyens JP , Bauer JM , et al. Sarcopenia: European consensus on definition and diagnosis: report of the European Working Group on Sarcopenia in Older People. Age Ageing. 2010;39(4):412‐423. 20392703 10.1093/ageing/afq034 PMC2886201

[6] Cruz‐Jentoft AJ , Sayer AA . Sarcopenia. Lancet. 2019;393(10191):2636‐2646. 31171417 10.1016/S0140-6736(19)31138-9

[7] Carvalho do Nascimento PR , Bilodeau M , Poitras S . How do we define and measure sarcopenia? A meta‐analysis of observational studies. Age Ageing. 2021;50(6):1906‐1913. doi:10.1093/ageing/afab148 34537833

[8] Petermann‐Rocha F , Balntzi V , Gray SR , et al. Global prevalence of sarcopenia and severe sarcopenia: a systematic review and meta‐analysis. J Cachexia Sarcopenia Muscle. 2022;13(1):86‐99. 34816624 10.1002/jcsm.12783 PMC8818604

[9] Tsekoura M , Kastrinis A , Katsoulaki M , Billis E , Gliatis J . Sarcopenia and its impact on quality of life. Adv Exp Med Biol. 2017;987:213‐218. 28971460 10.1007/978-3-319-57379-3_19

[10] Yuan S , Larsson SC . Epidemiology of sarcopenia: prevalence, risk factors, and consequences. Metabolism. 2023;144:155533. 36907247 10.1016/j.metabol.2023.155533

[11] Williams GR , Dunne RF , Giri S , Shachar SS , Caan BJ . Sarcopenia in the older adult with cancer. J Clin Oncol. 2021;39(19):2068‐2078. 34043430 10.1200/JCO.21.00102 PMC8260902

[12] Sun MY , Chang CL , Lu CY , Wu SY , Zhang JQ . Sarcopenia as an independent risk factor for specific cancers: a propensity score‐matched Asian population‐based cohort study. Nutrients. 2022;14(9):1910. 35565877 10.3390/nu14091910 PMC9105218

[13] Kim YM , Kim JH , Baik SJ , Chun J , Youn YH , Park H . Sarcopenia and sarcopenic obesity as novel risk factors for gastric carcinogenesis: a health checkup cohort study. Front Oncol. 2019;9:1249. 31799199 10.3389/fonc.2019.01249 PMC6868021

[14] Bano G , Trevisan C , Carraro S , et al. Inflammation and sarcopenia: a systematic review and meta‐analysis. Maturitas. 2017;96:10‐15. 28041587 10.1016/j.maturitas.2016.11.006

[15] Giovannini S , Brau F , Forino R , et al. Sarcopenia: diagnosis and management, state of the art and contribution of ultrasound. J Clin Med. 2021;10(23):5552. 34884255 10.3390/jcm10235552 PMC8658070

[16] Sudlow C , Gallacher J , Allen N , et al. UK biobank: an open access resource for identifying the causes of a wide range of complex diseases of middle and old age. PLoS Med. 2015;12(3):e1001779. 25826379 10.1371/journal.pmed.1001779 PMC4380465

[17] Arnold CM , Warkentin KD , Chilibeck PD , Magnus CR . The reliability and validity of handheld dynamometry for the measurement of lower‐extremity muscle strength in older adults. J Strength Cond Res. 2010;24(3):815‐824. 19661831 10.1519/JSC.0b013e3181aa36b8

[18] Dodds RM , Granic A , Robinson SM , Sayer AA . Sarcopenia, long‐term conditions, and multimorbidity: findings from UK Biobank participants. J Cachexia Sarcopenia Muscle. 2020;11(1):62‐68. 31886632 10.1002/jcsm.12503 PMC7015236

[19] Syddall HE , Westbury LD , Cooper C , Sayer AA . Self‐reported walking speed: a useful marker of physical performance among community‐dwelling older people? J Am Med Dir Assoc. 2015;16(4):323‐328. 25523286 10.1016/j.jamda.2014.11.004 PMC6600869

[20] Razali NM , Wah YB . Power comparisons of Shapiro‐Wilk, Kolmogorov‐Smirnov, Lilliefors and Anderson‐Darling tests. Analytics. 2011;2(1):21‐33.

[21] Efron B . The efficiency of Cox's likelihood function for censored data. J Am Stat Assoc. 1977;72(359):557‐565.

[22] Benjamini Y , Hochberg Y . Controlling the false discovery rate: a practical and powerful approach to multiple testing. J R Stat Soc Series B Stat Methodol. 1995;57(1):289‐300. doi:10.1111/j.2517-6161.1995.tb02031.x

[23] Bartsch H , Nair J . Chronic inflammation and oxidative stress in the genesis and perpetuation of cancer: role of lipid peroxidation, DNA damage, and repair. Langenbecks Arch Surg. 2006;391(5):499‐510. 16909291 10.1007/s00423-006-0073-1

[24] Listrat A , Lebret B , Louveau I , et al. How muscle structure and composition influence meat and flesh quality. Sci World J. 2016;2016:3182746. 10.1155/2016/3182746 PMC4789028 27022618

[25] Merz KE , Thurmond DC . Role of skeletal muscle in insulin resistance and glucose uptake. Compr Physiol. 2020;10(3):785‐809. 32940941 10.1002/cphy.c190029 PMC8074531

[26] Crawley DJ , Holmberg L , Melvin JC , et al. Serum glucose and risk of cancer: a meta‐analysis. BMC Cancer. 2014;14:985. 25526881 10.1186/1471-2407-14-985 PMC4320469

[27] Huang Y , Cai X , Qiu M , et al. Prediabetes and the risk of cancer: a meta‐analysis. Diabetologia. 2014;57(11):2261‐2269. 25208757 10.1007/s00125-014-3361-2

[28] Yang X , Bi P , Kuang S . Fighting obesity: when muscle meets fat. Adipocyte. 2014;3(4):280‐289. 26317052 10.4161/21623945.2014.964075 PMC4550683

[29] McPherron AC , Guo T , Bond ND , Gavrilova O . Increasing muscle mass to improve metabolism. Adipocyte. 2013;2(2):92‐98. 23805405 10.4161/adip.22500 PMC3661116

[30] Lengyel E , Makowski L , DiGiovanni J , Kolonin MG . Cancer as a matter of fat: the crosstalk between adipose tissue and tumors. Trends Cancer. 2018;4(5):374‐384. 29709261 10.1016/j.trecan.2018.03.004 PMC5932630

[31] Rogeri PS , Gasparini SO , Martins GL , et al. Crosstalk between skeletal muscle and immune system: which roles do IL‐6 and glutamine play? Front Physiol. 2020;11:582258. 33178046 10.3389/fphys.2020.582258 PMC7596683

[32] Shurin MR . Cancer as an immune‐mediated disease. Immunotargets Ther. 2012;1:1‐6. 27471681 10.2147/ITT.S29834 PMC4934149

[33] Parra‐Soto S , Pell JP , Celis‐Morales C , Ho FK . Absolute and relative grip strength as predictors of cancer: prospective cohort study of 445 552 participants in UK Biobank. J Cachexia Sarcopenia Muscle. 2022;13(1):325‐332. 34953058 10.1002/jcsm.12863 PMC8818619

[34] Jeong SM , Lee DH , Giovannucci EL . Predicted lean body mass, fat mass and risk of lung cancer: prospective US cohort study. Eur J Epidemiol. 2019;34(12):1151‐1160. 31754943 10.1007/s10654-019-00587-2 PMC7504685

[35] He Q , Xia B , Liu A , et al. Association of body composition with risk of overall and site‐specific cancers: a population‐based prospective cohort study. Int J Cancer. 2021;149(7):1435‐1447. 34019699 10.1002/ijc.33697

[36] Salerno EA , Saint‐Maurice PF , Willis EA , Moore SC , DiPietro L , Matthews CE . Ambulatory function and mortality among cancer survivors in the NIH‐AARP diet and health study. Cancer Epidemiol Biomarkers Prev. 2021;30(4):690‐698. 33664017 10.1158/1055-9965.EPI-20-1473 PMC8300589

[37] Abellan van Kan G , Rolland Y , Andrieu S , et al. Gait speed at usual pace as a predictor of adverse outcomes in community‐dwelling older people an international academy on nutrition and aging (IANA) task force. J Nutr Health Aging. 2009;13(10):881‐889.19924348 10.1007/s12603-009-0246-z

[38] Prado CM , Lieffers JR , McCargar LJ , et al. Prevalence and clinical implications of sarcopenic obesity in patients with solid tumours of the respiratory and gastrointestinal tracts: a population‐based study. Lancet Oncol. 2008;9(7):629‐635. doi:10.1016/S1470-2045(08)70153-0 18539529

[39] Link JC , Reue K . Genetic basis for sex differences in obesity and lipid metabolism. Annu Rev Nutr. 2017;37:225‐245. 28628359 10.1146/annurev-nutr-071816-064827 PMC5759759

[40] Winkler TW , Justice AE , Graff M , et al. The influence of age and sex on genetic associations with adult body size and shape: a large‐scale genome‐wide interaction study. PLoS Genet. 2015;11(10):e1005378. 26426971 10.1371/journal.pgen.1005378 PMC4591371

[41] Janssen I , Heymsfield SB , Wang ZM , Ross R . Skeletal muscle mass and distribution in 468 men and women aged 18‐88 yr. J Appl Physiol (1985). 2000;89(1):81‐88. 10904038 10.1152/jappl.2000.89.1.81

[42] Nuzzo JL . Narrative review of sex differences in muscle strength, endurance, activation, size, fiber type, and strength training participation rates, preferences, motivations, injuries, and neuromuscular adaptations. J Strength Cond Res. 2023;37(2):494‐536. 36696264 10.1519/JSC.0000000000004329

[43] Lundsgaard AM , Kiens B . Gender differences in skeletal muscle substrate metabolism – molecular mechanisms and insulin sensitivity. Front Endocrinol (Lausanne). 2014;5:195. 25431568 10.3389/fendo.2014.00195 PMC4230199

[44] Dent E , Morley JE , Cruz‐Jentoft AJ , et al. International clinical practice guidelines for sarcopenia (ICFSR): screening, diagnosis and management. J Nutr Health Aging. 2018;22(10):1148‐1161. 30498820 10.1007/s12603-018-1139-9 PMC12280515

