# Supplementary material for: Associations of sarcopenia, sarcopenia components and sarcopenic obesity with cancer incidence: A prospective cohort study of 414,094 participants in UK Biobank
Source: Int J Cancer. 2025 May 21;157(7):1316–32. doi: 10.1002/ijc.35480 (PMC12334911; doi:10.1002/ijc.35480)
Supplement: Supplementary file 1 — APPENDIX S1. Supporting information. [file IJC-157-1316-s001.pdf]

Supplementary Material

Associations of sarcopenia, sarcopenia components and sarcopenic obesity with cancer incidence: a prospective cohort study of 414,094 participants in UK Biobank

Panagiotis Filis, Christos K. Papagiannopoulos, Georgios Markozannes, Christos V. Chalitsios, Ioannis Zerdes, Antonios Valachis, Christopher Papandreou, Sofia Christakoudi, Konstantinos K. Tsilidis

Supplementary Text (pages 4-10)

Supplementary Text 1. Definition of Covariates.

Supplementary Text 2. Definition of prevalent and incident cancer.

Supplementary Text 3. Exclusion criteria definition.

Supplementary Tables (pages 11-24)

Supplementary Table 1: Incident cancer cases by sarcopenia and sex.

Supplementary Table 2. Incident Cancer Cases by Sarcopenia Components stratified by sex.

Supplementary Table 3. Association between individuals with probable/confirmed/severe sarcopenia versus without sarcopenia for total and site-specific cancer incidence, overall and by sex. Data are presented as hazard ratios and 95% confidence intervals. Abbreviations: FDR, False Discovery Rate; HR, Hazard Ratio; CI, Confidence Interval

Supplementary Table 4. Sensitivity analysis: Association between individuals with probable/confirmed/severe versus without sarcopenia for total and site-specific cancer incidence, overall and by sex after excluding 9,230 participants (F=4,335, M=4,895) who experienced any cancer type or death within the first 2 years of follow-up. Data are presented as hazard ratios and 95% confidence intervals. Abbreviations: FDR, False Discovery Rate; HR, Hazard Ratio; CI, Confidence Interval

Supplementary Table 5. Association between individuals with probable sarcopenia versus without sarcopenia for total and site-specific cancer incidence, overall and by sex. Data are presented as hazard ratios and 95% confidence intervals. Abbreviations: FDR, False Discovery Rate; HR, Hazard Ratio; CI, Confidence Interval

Supplementary Table 6. Sensitivity analysis: Association between individuals with probable sarcopenia versus without sarcopenia for total and site-specific cancer incidence, overall and by sex after excluding 9,230 participants (F=4,335, M=4,895) who experienced any cancer type or death within the first 2 years of follow-up. Data are presented as hazard ratios and 95% confidence intervals. Abbreviations: FDR, False Discovery Rate; HR, Hazard Ratio; CI, Confidence Interval

Supplementary Table 7. Linear association for continuous grip strength with total and site-specific cancer incidence, overall and by sex. Data are presented as hazard ratios and 95% confidence intervals. Abbreviations: FDR, False Discovery Rate; HR, Hazard Ratio; CI, Confidence Interval

Supplementary Table 8. Association between low muscle mass index versus normal muscle mass index for total and site-specific cancer incidence, overall and by sex. Data are presented as hazard ratios and 95% confidence intervals. Abbreviations: FDR, False Discovery Rate; HR, Hazard Ratio; CI, Confidence Interval

Supplementary Table 9. Sensitivity analysis: Association between low muscle mass index versus normal muscle mass index for total and site-specific cancer incidence, overall and by sex after excluding 9,230 participants (F=4,335, M=4,895) who experienced any cancer type or death within the first 2 years of follow-up. Data are presented as hazard ratios and 95% confidence intervals. Abbreviations: FDR, False Discovery Rate; HR, Hazard Ratio; CI, Confidence Interval

Supplementary Table 10. Linear association index for continuous muscle mass index (MMI) with total and site-specific cancer incidence, overall and by sex. Data are presented as hazard ratios and 95% confidence intervals. Abbreviations: FDR, False Discovery Rate; HR, Hazard Ratio; CI, Confidence Interval

Supplementary Table 11. Association between slow walking pace versus normal walking pace for total and site-specific cancer incidence, overall and by sex. Data are presented as hazard ratios and 95% confidence intervals. Abbreviations: FDR, False Discovery Rate; HR, Hazard Ratio; CI, Confidence Interval

Supplementary Table 12. Sensitivity analysis: Association between slow waking pace versus normal walking pace for total and site-specific cancer incidence, overall and by sex after excluding 9,230 participants (F=4,335, M=4,895) who experienced any cancer type or death within the first 2 years of follow-up. Data are presented as hazard ratios and 95% confidence intervals. Abbreviations: FDR, False Discovery Rate; HR, Hazard Ratio; CI, Confidence Interval

Supplementary Table 13. Association between sarcopenic obesity versus non-sarcopenic obesity for total and site-specific cancer incidence, overall and by sex. Data are presented as hazard ratios and 95% confidence intervals. Abbreviations: FDR, False Discovery Rate; HR, Hazard Ratio; CI, Confidence Interval

Supplementary Table 14. Sensitivity analysis: Association between sarcopenic obesity versus non-sarcopenic obesity for total and site-specific cancer incidence, overall and by sex after excluding 9,230 participants (F=4,335, M=4,895) who experienced any cancer type or death within the first 2 years of follow-up. Data are presented as hazard ratios and 95% confidence intervals. Abbreviations: FDR, False Discovery Rate; HR, Hazard Ratio; CI, Confidence Interval

Supplementary Figures (pages 25-28)

Supplementary Figure 1: Number of cancer cases in UK Biobank recorded from UK registries between 2018 and 2022.

Supplementary Figure 2. Percentages of Covariates missing values.

Supplementary Figure 3. Model 2 association of relative grip strength with cancer risk stratified by sex allowing for nonlinear effects. Reference is 23.59 (6.16) and 39.88 (8.69) mean (SD) value for females/males, respectively. Separate models were fitted for each cancer type, each with a restricted cubic spline for body composition (knots placed at 5th, 35th, 65th and 95th centiles). Red and blue colors represent females/males respectively.

Supplementary Figure 4. Model 2 association of relative muscle mass index (kg) with cancer risk stratified by sex allowing for nonlinear effects. Reference is 6.95(0.82) and 8.74(1.05) mean(SD) value for females/males, respectively. Separate models were fitted for each cancer type, each with a restricted cubic spline for body composition (knots placed at 5th, 35th, 65th and 95th centiles). Red and blue colours represent females/males respectively.

Supplementary References (page 29)

Supplementary Texts

Supplementary Text 1. Definition of Covariates.

<u>**Dietary Intake (DI) score**</u> was derived as described below.[1]

Category	Data Field	Variable	Description	Score	Condition
**Vegetables & Fruits**	1289.0.0	Cooked vegetable intake	On average how many heaped tablespoons of COOKED vegetables would you eat per DAY	1289.0.0/2 + 1299.0.0/2 + 1309.0.0 + 1319.0.0	1: Score < 5<br>0: Score >= 5
	1299.0.0	Salad / raw vegetable intake	On average how many heaped tablespoons of SALAD or RAW vegetables would you eat per DAY? (Include lettuce, tomato in sandwiches; put '0' if you do not eat any)		
	1309.0.0	Fresh fruit intake	About how many pieces of FRESH fruit would you eat per DAY? (Count one apple, one banana, 10 grapes etc as one piece; put '0' if you do not eat any		
	1319.0.0	Dried fruit intake	About how many pieces of DRIED fruit would you eat per DAY? (Count one prune, one dried apricot, 10 raisins as one piece; put '0' if you do not eat any)		
**Oily & Non-Oily Fishes**	1329.0.0	Oily fish intake	How often do you eat oily fish? (e.g. sardines, salmon, mackerel, herring)	1329.0.0 + 1339.0.0	1: Score = 0<br>0: Score > 0
	1339.0.0	Non-oily fish intake	How often do you eat other types of fish? (e.g. cod, tinned tuna, haddock)		
**Processed Meat**	1349.0.0	Processed meat intake	How often do you eat processed meats (such as bacon, ham, sausages, meat pies, kebabs, burgers, chicken nuggets)?	1349.0.0	1: Score > 2<br>0: Score <= 2
**Red Meat**	1369.0.0	Beef intake	How often do you eat pork/mutton? (Do not count processed meats)	1369.0.0 + 1379.0.0 + 1389.0.0	1: Score > 2<br>0: Score <= 2
	1379.0.0	Lamb/mutton intake	How often do you eat lamb/mutton? (Do not count processed meats)		
	1389.0.0	Pork intake	How often do you eat pork? (Do not count processed meats such as bacon or ham)		
**Milk**	1418.0.0	Milk type used	What type of milk do you mainly use	1418.0.0<br>1: Full cream, 2: Semi-skimmed, 3: Skimmed, 4: Soya, 5: Other type of milk, 6: Never/rarely have milk	1: Score = (1,5,6)<br>0: Score = (2,3,4)
**Spread**	1428.0.0	Spread type	What type of spread do you mainly use?	1428.0.0<br>1: Butter/spreadable butter, 2: Flora ProActive/Benecol, 3: Other type of spread/margarine, 0: Never/rarely use spread	1: Score = (1,2,3)<br>0: Score = 0
**Cereal**	1458.0.0	Cereal intake	How many bowls of cereal do you eat a WEEK?	1458.0.0	1: Score <= 5<br>0: Score > 5
**Salt**	1468.0.0	Salt added to food	Do you add salt to your food? (Do not include salt used in cooking)	1468.0.0<br>1: Never/rarely, 2: Sometimes, 3: Usually, 4: Alway	1: Score = (2,3,4)<br>0: Score = 1
**Water**	1478.0.0	Water intake	How many glasses of water do you drink each DAY?	1478.0.0	1: Score < 6<br>0: Score >= 6

Values greater than 50 replaced with missing. Values -10: “Less than one” replaced with 0.5. Missing values at each Dietary Intake were not ignored. DI derived summarizing the condition column. Higher values indicate an unhealthier DI.

Family History of Cancer was derived using Fields “Illnesses of father” [20107.0.0/9], Fields “Illnesses of mother” [20110.0.0/10] and Fields “Illnesses of siblings” [20111.0.0/11].
Values -11: “Do not know (group 1)”, -13 “Prefer not to answer (group 1)”, -17: ”None of the above (group 1)”. -21: “Do not know (group 2)”, -23: “Prefer not to answer (group 2)”, -27: “None of the above (group 2)” replaced with missing.
If values 5: “Breast cancer”, 4: ”Bowel cancer”, 3: ”Lung cancer”, 13: ” Prostate cancer” observed in any of the above Fields, participant grouped as 1: “Yes” else as 0: “No”.

Sedentary Behavior was derived by summing up the hours corresponding to each of the Fields “Time spent watching television” [1070.0.0], Fields “Time spent using computer” [1080.0.0] and Fields “Time spent driving” [1090.0.0]. Values greater than 50 replaced with missing. Values -10: “Less than one” replaced with 0.

Nonsteroidal anti-inflammatory drugs (NSAID) are defined as previously described in the literature. [2]

Physical Activity was derived by summing up the minutes per week of the Fields “MET per week for moderate activity” [22038.0.0], Fields “MET per week for vigorous activity” [22039.0.0] and Fields “MET minutes per week for walking” [22037.0.0].

Smoking Status was derived from Field “Smoking Status” [20116.0.0].

Smoking Intensity was derived from Field “Pack years of smoking” [20161.0.0]. Was calculated as the number of cigarettes per day / 20 * (Age stopped smoking - Age start smoking). If Field “Smoking Status” [20161.0.0] was 0 “Never”, smoke intensity was replaced with 0. If Field “Smoking Status” was 1 “Previous” or 2 ”Current”, smoke intensity was replaced with the median value of the observed part for each category respectively. In any other circumstances, smoke intensity replaced with missing.

Hormone Replacement Therapy: was derived from Field “Have you ever used hormone replacement therapy (HRT)” [2814.0.0]. Field [6153-0/3] “Medication for cholesterol, blood pressure, diabetes, or take exogenous hormones”, Answer 4 “Hormone Replacement Therapy” coded as “Yes”.

Diabetes status is defined as described previously in the literature using self-reported diabetes status, relevant medication questions, and ICD10 codes for diabetes.[2]

Townsend deprivation index as described in the literature.[3]

Cardiovascular status was studies as described in the literature.[4,5] Cardiovascular status was derived after re-classifying as category Yes participants with codes “1002”, “1081”, “1583”, “1082”, “1491”, “1086”, “1070” , ”6150” , “1076” , ”1074”, “1075”, “1095”, “1523” from Fields “Treatment/medication code2” [20003-0/47]. Furthermore, participants with ICD10 codes "I516", "I519", "I200", "I201", "I202", "I203", "I204", "I205", "I206", "I207", "I208", "I209", "I210", "I211", "I212", "I213", "I214", "I215", "I216", "I217", "I218", "I219", "I220", "I221", "I222", "I223", "I224", "I225", "I226", "I227", "I228", "I229", "I230", "I231", "I232", "I233", "I234", "I235", "I236", "I237", "I238", "I239", "I240", "I241", "I242", "I243", "I244", "I245", "I246", "I247", "I248", "I249", "I250", "I251", "I252", "I253", "I254", "I255", "I256", "I257", "I258", "I259", "I600", "I601", "I602", "I603", "I604", "I605", "I606", "I607", "I608", "I609", "I610", "I611", "I612", "I613", "I614", "I615", "I616", "I617", "I618", "I619", "I620", "I621", "I622", "I623", "I624", "I625", "I626", "I627", "I628", "I629", "I630", "I631", "I632", "I633", "I634", "I635", "I636", "I637", "I638", "I639", "I640", "I641", "I642", "I643", "I644", "I645", "I646", "I647", "I648", "I649", "G450", "G451", "G452", "G453", "G454", "G455", "G456", "G457", "G458", "G459", "I110", "I111", "I112", "I113", "I114", "I115", "I116", "I117", "I118", "I119", "I130", "I131", "I132", "I133", "I134", "I135", "I136", "I137", "I138", "I139", "I500", "I501", "I502", "I503", "I504", "I505", "I506", "I507", "I508", "I509", "I420", "I427", "I429", "I700", "I701", "I702", "I703", "I704", "I705", "I706", "I707", "I708", "I709", ”I710", "I711", "I712", "I713", "I714", "I715", "I716", "I717", "I718", "I719", "I720", "I721", "I722", "I723", "I724", "I725", "I726", "I727", "I728", "I729", "I730", "I731", "I732", "I733", "I734", "I735", "I736", "I737", "I738", "I739" and ICD9 codes 4292", "4100", "4101", "4102", "4103", "4104", "4105", "4106", "4107", "4108", "4109", "4110", "4111", "4112", "4113", "4114", "4115", "4116", "4117", "4118", "4119", "4120", "4121", "4122", "4123", "4124", "4125", "4126", "4127", "4128", "4129", "4140", "4141", "4142", "4143", "4144", "4145", "4146", "4147", "4148", "4149", "4340", "4341", "4342", "4343", "4344", "4345", "4346", "4347", "4348", "4349", "4350", "4351", "4352", "4353", "4354", "4355", "4356", "4357", "4358", "4359", "4310", "4311", "4312", "4313", "4314", "4315", "4316", "4317", "4318", "4319", "4300", "4301", "4302", "4303", "4304", "4305", "4306", "4307", "4308", "4309", "4280", "4281", "4282", "4283", "4284", "4285", "4286", "4287", "4288", "4289" grouped as diabetic from Field “Diagnoses - main ICD10” [41202.0.0/79] and Field “Diagnoses - main ICD9” [41203.0.0/27] respectively.

Oral contraceptive use was determined for women based on Field [2784-0.0] “Ever taken oral contraceptive pill”; Question: "Have you ever taken the contraceptive pill? (include the 'minipill')". Values -1: “Do not know”, -3: ”Prefer not to answer” replaced with missing.

Menopausal status was derived from Field "Had menopause" [2724.0.0]. Values 2: "Not sure - had a hysterectomy", 3: " Not sure - other reason" replaced with missing.

Mammogram status was derived from Field "Ever had breast cancer screening / mammogram" [2674.0.0].

Comorbidity status was a binary variable and was 1: if participants had self-reported at enrollment any of the attached:

1) liver-related non-cancer illness or kidney failure
2) inflammatory bowel disease
3) chronic respiratory non-cancer illness
4) heart failure

Participants with no records of the aforementioned determined as 0. More information about UK Biobank fields and used codes can be found elsewhere.[2]

At each covariate values, -3: "Prefer not to answer" and -1: "Do not know" replaced with missing. Missing values were excluded from the analysis (**Supplementary Figure 2**).

Supplementary Text 2. Definition of prevalent and incident cancer

Definition of prevalent and incident cancer was based on previous literature.[6]

Information on prevalent cancers was obtained from the cancer registry and self-reported cancer. Information for incident cancers was obtained from the cancer registry. For the cancer registry, information was included in Fields [40005-0.0/21] "Date of cancer diagnosis"; Fields [40006-0.0/21] "Type of cancer: ICD10", Fields [40013-0.0/21] "Type of cancer: ICD9" (9th version of ICD, relevant only to prevalent cancers), Fields [40011-0.0/21] "Histology of cancer tumour" and Fields [40012-0.0/21] "Behaviour of cancer tumour". Many participants in UKB had been diagnosed with numerous cancers, with a maximum value of 21 records. Each cancer had its respective date of diagnosis, ICD10 or ICD9, morphology and histology. We restricted participants with multiple cancer diagnoses separately, covering the cancer registry fields in a long format. We excluded entries if:

1) There was missing information for the date of diagnosis.

2) There were non-malignant cancer types, i.e. did not begin with "C" for ICD10 coding.

3) There were non-melanoma skin cancers (defined with code 173 for ICD9 or C44 for ICD10), except skin cancers with squamous cell morphology (codes 8070, 8071, 8072 or 8083 for histology).

4) Tumors with topological code corresponding to malignant cancer (140 to 209 for ICD9 or beginning with "C" for ICD10) but with behavioural codes 0, 1 or 2, as these are similar, correspondingly, to tumours with codes 210-229, 235-239 and 230-234 for ICD9 or tumours with codes D10-D36, D37-D48 and D00-D09 for ICD10.

Incident cancer was considered present if there was an entry in the cancer registry with a date of diagnosis after the date of attending an assessment centre at baseline. Cancers with morphology codes 3 "Malignant, primary site" or 5 "Malignant, micro-invasive" were considered cases. Participants with first incident cancer with behavioural code 6 "Malignant, metastatic site", 9 "Malignant, uncertain whether primary or metastatic site" or missing were censored at the date of cancer diagnosis. Morphology types 0:"Benign", 1:" Uncertain whether benign or malignant", and 2:"Carcinoma in situ" were censored at the date of diagnosis and allowed to continue follow-up until the other codes were recorded. If there were only 0:"Benign", 1:" Uncertain whether benign or malignant", or 2:"Carcinoma in situ", we kept as censor the most recent date of diagnosis.

In total, there were 109,689 participants out of which 72,584 had malignant cancer diagnosis.

Supplementary Text 3. Exclusion criteria.

We excluded individuals with late or incomplete dates from cancer registries (N=2,324), non-white ancestry (N=28,336), aneuploidy (this indicates samples which were identified as putatively carrying sex chromosome configurations that are not either XX or XY) or if there was a mismatch between the self-reported and biological sex (N=801). To minimize reverse causation, we further excluded participants with prevalent cancer diagnosed at baseline (N=35,947), rare cancer histologies (N=325), or self-reported cancers at baseline (N=6,563). Prevalent comorbidities were not excluded, but instead were used as part of the adjustments. Participants with missing values in any sarcopenia components were also excluded (N=11,910). We further excluded those with extreme (height ≤ 130 cm, BMI ≤ 15 kg/m^2 or ≥ 60 kg/m^2) anthropometric measurements (N=59).

The exclusion criteria were applied sequentially in the displayed order. Specific fields used to define the exclusions are listed below:

1) Data from the registries is sent to UK Biobank periodically and then incorporated into the Resource. This, along with delays occurring before the record appears in the relevant cancer registry (usually at least 6 months and sometimes much longer) and processing time, means that data is not typically available to researchers until at least a year after diagnosis, and in some cases much longer. Later dates than "**31-12-2020**" were characterized as late or incomplete (Supplementary Figure 1).[7,8]

2) Only white European participants were included because of the ethnic differences in the reference values for sarcopenia.[9]
Field [21000-0.0] "Ethnic background"; in the study were retained participants with codes: 1 "White", 1001 "British", 1002 "Irish", 1003 "Any other white background".

3) Participants with code 1 for Field [22019-0.0] "Sex chromosome aneuploidy" OR with a mismatch between Field [22001-0.0] "Genetic sex" and Field [34-0.0] "Sex (self-reported)".

4) Prevalent cancer was considered present if its respective date was earlier or up to the initial assessment date.

5) Rare cancer histologies excluded, as previously decribed.[6,10]

6) Information on self-reported prevalent cancers was obtained from Fields [20001-0.0/5] “Cancer code, self-reported.” Prevalent cancer was considered present if there was a self-reported cancer at baseline.

7) Sarcopenia components were obtained from Fields [46-0.0] “Hand grip strength (left)”, Field [47-0.0] “Hand grip strength (right)”, Field [23121-0.0] “Arm fat-free mass (right)”, field [23125-0.0] “Arm fat-free mass (left)”, Field [23113-0.0] “Leg fat-free mass (right)”, Field [23117-0.0] “Leg fat-free mass (left)”, Field [50-0.0] “Standing Height” and Field [924-0.0] “Usual walking pace”.

8) Anthropometric measurements were obtained from Field [21002-0.0] “Weight” composing BMI (Weight/Height^2). Very extreme values were BMI $\leq$ 15 kg/m^2 or BMI $\geq$ 60 kg/m^2, as well as height < 130cm. Participants with missing weight were removed.

9a) exclusions comprised hysterectomy self-reported at enrolment with codes:
1357 hysterectomy
1358 hysterectomy with oophorectomy
1359 hysterectomy with cervical sparing

9b) exclusions comprised oophorectomy self-reported at enrolment with codes:
1355 bilateral oophorectomy

Supplementary Tables

Supplementary Table 1. Incident cancer cases by sarcopenia categories stratified by sex.

Cancer		Overall (F = 222, 516 M = 191, 578)	Non-Sarcopenic (F= 201, 091, M= 179, 783)	Probable Sarcopenia (F= 21, 186, M= 11, 100)	Confirmed and Severe Sarcopenia (F=239, M=695)
**Oral**	F	244 (0.1)	224 (0.1)	20 (0.1)	0 (0)
	M	494 (0.3)	454 (0.3)	35 (0.3)	5 (0.7)
**Esophageal**	F	222 (0.1)	198 (0.1)	23 (0.1)	1 (0.4)
	M	580 (0.3)	527 (0.3)	52 (0.5)	1 (0.1)
**Gastric**	F	168 (0.1)	149 (0.1)	19 (0.1)	0 (0)
	M	358 (0.2)	331 (0.2)	26 (0.2)	1 (0.1)
**Colorectal**	F	1,898 (0.9)	1,676 (0.8)	219 (1)	3 (1.3)
	M	2,373 (1.2)	2,174 (1.2)	192 (1.7)	7 (1)
**Liver**	F	170 (0.1)	143 (0.1)	26 (0.1)	1 (0.4)
	M	306 (0.2)	259 (0.1)	46 (0.4)	1 (0.1)
**Gallbladder**	F	57 (0)	50 (0)	7 (0)	0 (0)
	M	34 (0)	32 (0)	2 (0)	0 (0)
**Pancreatic**	F	456 (0.2)	399 (0.2)	57 (0.3)	0 (0)
	M	523 (0.3)	477 (0.3)	46 (0.4)	0 (0)
**Lung**	F	1,552 (0.7)	1,318 (0.7)	228 (1.1)	6 (2.5)
	M	1,582 (0.8)	1,429 (0.8)	138 (1.2)	15 (2.2)
**Hematologic**	F	1,476 (0.7)	1,296 (0.6)	180 (0.8)	0 (0)
	M	1,739 (0.9)	1,593 (0.9)	136 (1.2)	10 (1.4)
**Melanoma**	F	1,015 (0.5)	926 (0.5)	87 (0.4)	2 (0.8)
	M	881 (0.5)	830 (0.5)	49 (0.4)	2 (0.3)
**Breast**	F	7,403 (3.3)	6,725 (3.3)	675 (3.2)	3 (1.3)
**Cervical**	F	86 (0)	79 (0)	7 (0)	0 (0)
**Endometrial**	F	1,050 (0.5)	931 (0.5)	119 (0.6)	0 (0)
**Ovarian**	F	765 (0.3)	678 (0.3)	85 (0.4)	2 (0.8)
**Prostate**	M	523 (0.3)	477 (0.3)	46 (0.4)	0 (0)
**Kidney**	F	411 (0.2)	363 (0.2)	48 (0.2)	0 (0)
	M	697 (0.4)	658 (0.4)	38 (0.3)	1 (0.1)
**Bladder**	F	184 (0.1)	151 (0.1)	31 (0.1)	2 (0.8)
	M	547 (0.3)	504 (0.3)	36 (0.3)	7 (1)
**Brain**	F	284 (0.1)	252 (0.1)	31 (0.1)	1 (0.4)

	M	390 (0.2)	372 (0.2)	18 (0.2)	0 (0)
**Overall**	F	29,650 (13.3)	26,325 (13.1)	3,281 (15.5)	44 (18.4)
	M	33,729 (17.6)	31,302 (17.4)	2,278 (20.5)	149 (21.4)

Data are presented as Number (%)

Supplementary Table 2. Incident Cancer Cases by Sarcopenia Components stratified by sex.

Cancers		NS N=364,452	PS N=29,938	PS/S N=30,737	Normal MMI N=389,107	Low MMI N=6,082	Normal GS N=364,452	Low GS N=30,737	NS-O N=85,353	S-O N=9,133	Normal WP N=369,399	Slow WP N=25,790
Oral	F	224 (0.1)	20 (0.1)	20 (0.1)	243 (0.1)	1 (0.1)	224 (0.1)	20 (0.1)	48 (0.1)	4 (0.1)	217 (0.1)	27 (0.2)
	M	454 (0.3)	35 (0.3)	40 (0.3)	448 (0.2)	46 (0.9)	454 (0.3)	40 (0.3)	119 (0.3)	10 (0.3)	439 (0.2)	55 (0.4)
Oesophageal	F	198 (0.1)	23 (0.1)	24 (0.1)	212 (0.1)	10 (0.6)	198 (0.1)	24 (0.1)	41 (0.1)	9 (0.1)	201 (0.1)	21 (0.1)
	M	527 (0.3)	52 (0.5)	53 (0.4)	562 (0.3)	18 (0.3)	527 (0.3)	53 (0.4)	189 (0.4)	26 (0.7)	512 (0.3)	68 (0.5)
Gastric	F	149 (0.1)	19 (0.1)	19 (0.1)	167 (0.1)	1 (0.1)	149 (0.1)	19 (0.1)	39 (0.1)	7 (0.1)	152 (0.1)	16 (0.1)
	M	331 (0.2)	26 (0.2)	27 (0.2)	350 (0.2)	8 (0.2)	331 (0.2)	27 (0.2)	103 (0.2)	10 (0.3)	316 (0.2)	42 (0.3)
Colorectal	F	1,676 (0.8)	219 (1)	222 (1)	1,883 (0.9)	15 (0.9)	1,676 (0.8)	222 (1)	403 (0.9)	68 (1.1)	1,742 (0.8)	156 (1)
	M	2,174 (1.2)	192 (1.7)	199 (1.7)	2,302 (1.2)	71 (1.4)	2,174 (1.2)	199 (1.7)	653 (1.4)	78 (2.2)	2,145 (1.2)	228 (1.7)
Liver	F	143 (0.1)	26 (0.1)	27 (0.1)	168 (0.1)	2 (0.1)	143 (0.1)	27 (0.1)	44 (0.1)	8 (0.1)	147 (0.1)	23 (0.1)
	M	259 (0.1)	46 (0.4)	47 (0.4)	299 (0.2)	7 (0.1)	259 (0.1)	47 (0.4)	124 (0.3)	21 (0.6)	245 (0.1)	61 (0.5)
Gallbladder	F	50 (0)	7 (0)	7 (0)	57 (0)	0 (0)	50 (0)	7 (0)	18 (0)	4 (0.1)	46 (0)	11 (0.1)
	M	32 (0)	2 (0)	2 (0)	34 (0)	0 (0)	32 (0)	2 (0)	12 (0)	0 (0)	31 (0)	3 (0)
Pancreatic	F	399 (0.2)	57 (0.3)	57 (0.3)	453 (0.2)	3 (0.2)	399 (0.2)	57 (0.3)	117 (0.3)	22 (0.3)	409 (0.2)	47 (0.3)
	M	477 (0.3)	46 (0.4)	46 (0.4)	514 (0.3)	9 (0.2)	477 (0.3)	46 (0.4)	146 (0.3)	21 (0.6)	459 (0.3)	64 (0.5)
Lung	F	1,318 (0.7)	228 (1.1)	234 (1.1)	1,527 (0.7)	25 (1.6)	1,318 (0.7)	234 (1.1)	301 (0.7)	73 (1.1)	1,273 (0.6)	279 (1.7)
	M	1,429 (0.8)	138 (1.2)	153 (1.3)	1,466 (0.8)	116 (2.3)	1,429 (0.8)	153 (1.3)	393 (0.9)	45 (1.3)	1,268 (0.7)	314 (2.4)
Hematologic	F	1,296 (0.6)	180 (0.8)	180 (0.8)	1,468 (0.7)	8 (0.5)	1,296 (0.6)	180 (0.8)	317 (0.7)	50 (0.8)	1,357 (0.7)	119 (0.7)
	M	1,593 (0.9)	136 (1.2)	146 (1.2)	1,688 (0.9)	51 (1)	1,593 (0.9)	146 (1.2)	429 (1)	44 (1.2)	1,582 (0.9)	157 (1.2)
Melanoma	F	926 (0.5)	87 (0.4)	89 (0.4)	1,008 (0.5)	7 (0.4)	926 (0.5)	89 (0.4)	196 (0.4)	29 (0.5)	960 (0.5)	55 (0.3)
	M	830 (0.5)	49 (0.4)	51 (0.4)	860 (0.5)	21 (0.4)	830 (0.5)	51 (0.4)	225 (0.5)	19 (0.5)	834 (0.5)	47 (0.4)
Breast	F	6,725 (3.3)	675 (3.2)	678 (3.2)	7361 (3.3)	42 (2.6)	6,725 (3.3)	678 (3.2)	1,595 (3.6)	218 (3.4)	6838 (3.3)	565 (3.5)
Cervical	F	79 (0)	7 (0)	7 (0)	85 (0)	1 (0.1)	79 (0)	7 (0)	20 (0)	0 (0)	82 (0)	4 (0)
Endometrial	F	931 (0.5)	119 (0.6)	119 (0.6)	1049 (0.5)	1 (0.1)	931 (0.5)	119 (0.6)	429 (1)	61 (1)	914 (0.4)	136 (0.8)
Ovarian	F	678 (0.3)	85 (0.4)	87 (0.4)	759 (0.3)	6 (0.4)	678 (0.3)	87 (0.4)	167 (0.4)	27 (0.4)	698 (0.3)	67 (0.4)
Prostate	M	7,818 (4.3)	526 (4.7)	563 (4.8)	8,119 (4.4)	262 (5.1)	7818 (4.3)	563 (4.8)	1,735 (3.9)	147 (4.1)	7,866 (4.4)	515 (3.9)
Kidney	F	363 (0.2)	48 (0.2)	48 (0.2)	410 (0.2)	1 (0.1)	363 (0.2)	48 (0.2)	125 (0.3)	20 (0.3)	359 (0.2)	52 (0.3)
	M	658 (0.4)	38 (0.3)	39 (0.3)	686 (0.4)	11 (0.2)	658 (0.4)	39 (0.3)	237 (0.5)	20 (0.6)	623 (0.3)	74 (0.6)
Bladder	F	151 (0.1)	31 (0.1)	33 (0.2)	182 (0.1)	2 (0.1)	151 (0.1)	33 (0.2)	38 (0.1)	16 (0.2)	154 (0.1)	30 (0.2)
	M	504 (0.3)	36 (0.3)	43 (0.4)	520 (0.3)	27 (0.5)	504 (0.3)	43 (0.4)	154 (0.3)	13 (0.4)	484 (0.3)	63 (0.5)
Brain	F	252 (0.1)	31 (0.1)	32 (0.1)	282 (0.1)	2 (0.1)	252 (0.1)	32 (0.1)	50 (0.1)	10 (0.2)	265 (0.1)	19 (0.1)
	M	372 (0.2)	18 (0.2)	18 (0.2)	381 (0.2)	9 (0.2)	372 (0.2)	18 (0.2)	93 (0.2)	6 (0.2)	364 (0.2)	26 (0.2)
Overall	F	26,325 (13.1)	3,281 (15.5)	3,325 (15.5)	29,422 (13.3)	228 (14.2)	26,325 (13.1)	3,325 (15.5)	6,088 (13.7)	1,016 (15.9)	27,041 (13.1)	2,609 (16.2)
	M	31,302 (17.4)	2,278 (20.5)	2,427 (20.6)	32,565 (17.5)	1,164 (22.6)	31,302 (17.4)	2,427 (20.6)	7,762 (17.2)	716 (20.1)	30,912 (17.3)	2,817 (21.4)

Data are presented N(%). Abbreviations: GS, Grip Strength; MMI, Muscle Mass Index; NS, Non-Sarcopenic; NS-O, Non-Sarcopenic Obesity; PS, Probable Sarcopenia; S-O, Sarcopenic Obesity; S, Sarcopenia; WP, Walking Pace; F, Female; M, Male;

Supplementary Table 3. Association between individuals with probable/confirmed/severe sarcopenia versus without sarcopenia for total and site-specific cancer incidence, overall and by sex. Data are presented as hazard ratios and 95% confidence intervals. Abbreviations: FDR, False Discovery Rate; HR, Hazard Ratio; CI, Confidence Interval

Cancer	HR(95%CI)	P-Value	Female HR(95%CI)	P-Value	Male HR(95%CI)	P-Value	Interaction by sex P-Value	N of Cases (F / M)
**Model 1**								
**Oral**	1.07 (0.81, 1.42)	0.643	0.8 (0.49, 1.3)	0.361	1.27 (0.9, 1.79)	0.167	0.121	20 / 40
**Oesophageal**	0.98 (0.76, 1.27)	0.9	0.77 (0.49, 1.21)	0.254	1.12 (0.82, 1.52)	0.468	0.174	24 / 53
**Gastric**	0.97 (0.71, 1.33)	0.87	0.9 (0.56, 1.46)	0.683	1.03 (0.69, 1.54)	0.888	0.685	19 / 27
**Colorectal**	1.09 (0.98, 1.21)	0.113	0.98 (0.85, 1.14)	0.802	1.23 (1.06, 1.43)	**0.007***	**0.034**	222 / 199
**Liver**	1.54 (1.17, 2.02)	**0.002***	1.14 (0.73, 1.76)	0.565	1.92 (1.36, 2.7)	**<0.001***	0.064	27 / 47
**Pancreatic**	1.03 (0.82, 1.28)	0.805	0.97 (0.73, 1.3)	0.848	1.11 (0.8, 1.54)	0.538	0.554	57 / 46
**Lung**	1.06 (0.95, 1.19)	0.304	1.1 (0.95, 1.27)	0.218	1.01 (0.85, 1.21)	0.888	0.497	234 / 153
**Hematologic**	1.11 (0.98, 1.25)	0.104	1.03 (0.87, 1.21)	0.725	1.22 (1.02, 1.46)	**0.034**	0.178	180 / 146
**Melanoma**	0.88 (0.73, 1.06)	0.187	0.88 (0.7, 1.11)	0.269	0.89 (0.65, 1.22)	0.461	0.953	89 / 51
**Breast**	0.94 (0.86, 1.02)	0.134	0.94 (0.86, 1.02)	0.134				678
**Endometrial**	0.97 (0.79, 1.18)	0.745	0.97 (0.79, 1.18)	0.745				119
**Ovarian**	1.08 (0.85, 1.37)	0.516	1.08 (0.85, 1.37)	0.516				87
**Prostate**	0.95 (0.87, 1.04)	0.26			0.95 (0.87, 1.04)	0.26		563
**Kidney**	0.83 (0.66, 1.05)	0.116	0.92 (0.68, 1.26)	0.624	0.74 (0.52, 1.04)	0.082	0.338	48 / 39
**Bladder**	1.03 (0.79, 1.35)	0.811	1.36 (0.9, 2.05)	0.14	0.87 (0.61, 1.24)	0.435	0.102	33 /43
**Brain**	0.89 (0.66, 1.21)	0.462	1.09 (0.75, 1.59)	0.663	0.66 (0.39, 1.11)	0.114	0.123	32 / 18
**Overall**	0.99 (0.97, 1.02)	0.727	0.97 (0.94, 1.01)	0.126	1.03 (0.98, 1.08)	0.2	0.048	3325 / 2427
**Model 2**								
**Oral**	1.06 (0.8, 1.4)	0.686	0.8 (0.49, 1.29)	0.359	1.26 (0.89, 1.77)	0.193	0.131	18 / 37
**Oesophageal**	0.97 (0.75, 1.25)	0.806	0.76 (0.48, 1.2)	0.238	1.1 (0.81, 1.49)	0.548	0.186	21 / 46
**Gastric**	0.98 (0.71, 1.34)	0.885	0.91 (0.56, 1.47)	0.686	1.03 (0.69, 1.55)	0.871	0.678	19 / 26
**Colorectal**	1.08 (0.97, 1.2)	0.163	0.98 (0.84, 1.13)	0.755	1.21 (1.04, 1.41)	**0.013**	**0.044**	202 / 183
**Liver**	1.37 (1.04, 1.81)	**0.023**	1.07 (0.69, 1.65)	0.777	1.65 (1.17, 2.33)	**0.004**	0.12	24 / 39
**Pancreatic**	1.01 (0.81, 1.26)	0.916	0.98 (0.73, 1.31)	0.88	1.06 (0.76, 1.48)	0.73	0.718	52 / 38
**Lung**	1.01 (0.9, 1.13)	0.909	1.04 (0.9, 1.21)	0.564	0.95 (0.8, 1.14)	0.614	0.445	213 / 137
**Hematologic**	1.1 (0.98, 1.25)	0.113	1.03 (0.87, 1.21)	0.753	1.22 (1.01, 1.46)	**0.035**	0.173	165 / 130
**Melanoma**	0.89 (0.74, 1.07)	0.224	0.89 (0.7, 1.11)	0.297	0.9 (0.66, 1.23)	0.518	0.925	82 / 42
**Breast**	0.92 (0.84, 1.02)	0.102	0.92 (0.84, 1.02)	0.102				637
**Breast (Pre)**			1.18 (0.81, 1.71)	0.386				31
**Breast(Post)**			0.92 (0.84, 1)	**0.049**				601
**Endometrial**	0.98 (0.79, 1.21)	0.84	0.98 (0.79, 1.21)	0.84				108
**Ovarian**	1.07 (0.83, 1.39)	0.59	1.07 (0.83, 1.39)	0.59				82
**Prostate**	0.96 (0.87, 1.06)	0.434			0.96 (0.87, 1.06)	0.434		499
**Kidney**	0.8 (0.64, 1.01)	0.064	0.91 (0.67, 1.24)	0.557	0.7 (0.49, 0.99)	**0.042**	0.261	45 / 34
**Bladder**	1.01 (0.77, 1.32)	0.952	1.31 (0.87, 1.99)	0.198	0.86 (0.6, 1.22)	0.393	0.122	27 /34
**Brain**	0.9 (0.66, 1.22)	0.496	1.08 (0.74, 1.58)	0.678	0.67 (0.4, 1.13)	0.135	0.144	31 / 15
**Overall**	0.99 (0.96, 1.02)	0.372	0.97 (0.93, 1)	0.072	1.02 (0.97, 1.06)	0.45	0.079	3071 / 2148

Model 1: Adjusted for age, gender (female, male), BMI, Townsend deprivation index (TDI), smoking status (never/ former/current), alcohol frequency intake (never, 1-3 t/m, 1-2 t/w, >3 t/w), MET, family history of (No/Yes). **Model 2:** As model 1, plus 9-item dietary intake score, sedentary behaviour, cardiovascular disease (no/yes), diabetes (no/yes), NSAID (no/yes). For prostate cancer, we also adjusted for testosterone and SHBG concentrations. For breast cancer, we also adjusted for history of mammography (no/yes). Additional adjustments for female specific cancers were menopausal status (no/yes), oral contraceptive use (no/yes), HRT use (no/yes), and age at menarche. Pre: pre-menopausal, Post: post-menopausal.

*: FDR-significance

Supplementary Table 4. Sensitivity analysis: Association between individuals with probable/confirmed/severe versus without sarcopenia for total and site-specific cancer incidence, overall and by sex after excluding 9,230 participants (F=4,335, M=4,895) who experienced any cancer type or death within the first 2 years of follow-up. Data are presented as hazard ratios and 95% confidence intervals. Abbreviations: FDR, False Discovery Rate; HR, Hazard Ratio; CI, Confidence Interval

Cancer	HR(95%CI)	P-Value	Female HR(95%CI)	P-Value	Male HR(95%CI)	P-Value	Interaction by sex P-Value
**Oral**	0.99 (0.73, 1.35)	0.968	0.82 (0.5, 1.35)	0.438	1.13 (0.77, 1.66)	0.534	0.317
**Oesophageal**	1.06 (0.81, 1.38)	0.673	0.86 (0.54, 1.36)	0.513	1.19 (0.86, 1.64)	0.288	0.249
**Gastric**	0.96 (0.68, 1.34)	0.789	0.88 (0.53, 1.47)	0.626	1.02 (0.66, 1.58)	0.936	0.672
**Colorectal**	1.07 (0.95, 1.2)	0.243	0.97 (0.83, 1.14)	0.702	1.21 (1.02, 1.43)	**0.026**	0.058
**Liver**	1.37 (1.03, 1.83)	**0.03**	1.03 (0.65, 1.65)	0.898	1.67 (1.17, 2.38)	**0.005**	0.104
**Pancreatic**	1.05 (0.83, 1.32)	0.703	0.97 (0.71, 1.32)	0.842	1.16 (0.82, 1.64)	0.403	0.447
**Lung**	1.03 (0.91, 1.16)	0.623	1.09 (0.94, 1.28)	0.248	0.94 (0.78, 1.14)	0.541	0.227
**Hematologic**	1.1 (0.96, 1.25)	0.156	1.04 (0.87, 1.23)	0.668	1.19 (0.98, 1.44)	0.086	0.314
**Melanoma**	0.89 (0.73, 1.09)	0.27	0.9 (0.7, 1.15)	0.384	0.89 (0.63, 1.24)	0.491	0.965
**Breast**	0.93 (0.84, 1.03)	0.157	0.93 (0.84, 1.03)	0.157			
**Breast (Pre)**			1.01 (0.63, 1.67)	0.965			
**Breast(Post)**			0.92 (0.84, 1.01)	0.091			
**Endometrial**	1 (0.8, 1.26)	0.973	1 (0.8, 1.26)	0.973			
**Ovarian**	1.09 (0.82, 1.45)	0.538	1.09 (0.82, 1.45)	0.538			
**Prostate**	0.99 (0.89, 1.1)	0.804			0.99 (0.89, 1.1)	0.804	
**Kidney**	0.82 (0.64, 1.04)	0.107	0.97 (0.7, 1.34)	0.831	0.67 (0.46, 0.98)	**0.039**	0.153
**Bladder**	0.97 (0.72, 1.31)	0.849	1.24 (0.78, 1.98)	0.367	0.85 (0.58, 1.24)	0.387	0.21
**Brain**	0.88 (0.63, 1.23)	0.44	1.06 (0.7, 1.6)	0.783	0.64 (0.36, 1.15)	0.138	0.169
**Overall**	1 (0.97, 1.03)	0.834	0.97 (0.93, 1.01)	0.192	1.03 (0.98, 1.08)	0.218	0.072

Adjusted for age, gender (female, male), BMI, Townsend deprivation index (TDI), smoking status (never/ former/current), alcohol frequency intake (never, 1-3 t/m, 1-2 t/w, >3 t/w), MET, family history of (No/Yes), 9-item dietary intake, sedentary behavior, cardiovascular disease (no/yes), diabetes (no/yes), NSAID (no/yes). For Prostate we also adjusted for testosterone and SHBG. For breast cancer, we also adjusted for history of mammography (no/yes). Additional adjustments for female specific cancers were menopause status (no/yes), contraceptive use (no/yes), HRT (no/yes), and age at menarche. Pre: pre-menopausal, Post: post-menopausal.

*: FDR-significance.

Supplementary Table 5. Association between individuals with probable sarcopenia versus without sarcopenia for total and site-specific cancer incidence, overall and by sex. Data are presented as hazard ratios and 95% confidence intervals. Abbreviations: FDR, False Discovery Rate; HR, Hazard Ratio; CI, Confidence Interval

Cancer	HR(95%CI)	P-Value	Female HR(95%CI)	P-Value	Male HR(95%CI)	P-Value	Interaction by sex P-Value	N of Cases (F / M)
				**Model 1**				
**Oral**	1.02 (0.76, 1.37)	0.87	0.81 (0.5, 1.31)	0.389	1.2 (0.83, 1.72)	0.329	0.2	20 / 35
**Oesophageal**	0.99 (0.76, 1.28)	0.933	0.74 (0.46, 1.17)	0.197	1.15 (0.85, 1.57)	0.361	0.113	23 / 52
**Gastric**	0.99 (0.72, 1.35)	0.926	0.91 (0.56, 1.48)	0.713	1.04 (0.69, 1.58)	0.837	0.678	19 / 26
**Colorectal**	1.1 (0.99, 1.23)	0.077	0.98 (0.84, 1.13)	0.749	1.27 (1.09, 1.48)	**0.002***	**0.014**	219 / 192
**Liver**	1.52 (1.15, 2.01)	**0.003**	1.1 (0.7, 1.71)	0.685	1.94 (1.37, 2.75)	**<0.001***	**0.045**	26 / 46
**Pancreatic**	1.06 (0.85, 1.32)	0.631	0.98 (0.73, 1.31)	0.893	1.17 (0.84, 1.63)	0.355	0.432	57 / 46
**Lung**	1.06 (0.94, 1.19)	0.326	1.08 (0.93, 1.26)	0.284	1.02 (0.85, 1.23)	0.805	0.632	228 / 136
**Hematologic**	1.11 (0.98, 1.26)	0.1	1.04 (0.88, 1.23)	0.63	1.21 (1.01, 1.46)	**0.043**	0.228	180 / 138
**Melanoma**	0.88 (0.72, 1.06)	0.165	0.87 (0.69, 1.09)	0.222	0.89 (0.65, 1.23)	0.49	0.874	87 / 49
**Breast**	0.94 (0.87, 1.03)	0.18	0.94 (0.87, 1.03)	0.18				675
**Endometrial**	0.97 (0.79, 1.19)	0.774	0.97 (0.79, 1.19)	0.774				119
**Ovarian**	1.07 (0.84, 1.35)	0.603	1.07 (0.84, 1.35)	0.603				85
**Prostate**	0.95 (0.87, 1.05)	0.311			0.95 (0.87, 1.05)	0.311		526
**Kidney**	0.84 (0.66, 1.06)	0.139	0.93 (0.68, 1.27)	0.651	0.75 (0.53, 1.06)	0.098	0.351	48 / 38
**Bladder**	0.94 (0.7, 1.24)	0.644	1.27 (0.83, 1.94)	0.266	0.76 (0.52, 1.12)	0.16	0.076	31 / 36
**Brain**	0.9 (0.66, 1.23)	0.514	1.07 (0.73, 1.56)	0.746	0.7 (0.42, 1.17)	0.176	0.198	31 / 18
**Overall**	0.99 (0.97, 1.02)	0.71	0.97 (0.93, 1.01)	0.116	1.03 (0.99, 1.08)	0.181	**0.04**	3281 / 2278
				**Model 2**				
**Oral**	1.02 (0.76, 1.36)	0.911	0.81 (0.5, 1.31)	0.389	1.18 (0.82, 1.7)	0.367	0.215	18 / 32
**Oesophageal**	0.98 (0.75, 1.26)	0.851	0.73 (0.46, 1.16)	0.186	1.14 (0.83, 1.55)	0.422	0.12	20 / 45
**Gastric**	0.99 (0.72, 1.35)	0.938	0.91 (0.56, 1.48)	0.715	1.05 (0.69, 1.58)	0.823	0.671	19 / 25
**Colorectal**	1.09 (0.98, 1.22)	0.104	0.97 (0.84, 1.13)	0.703	1.26 (1.08, 1.47)	**0.003***	**0.016**	199 / 180
**Liver**	1.37 (1.04, 1.81)	**0.027**	1.03 (0.66, 1.61)	0.895	1.68 (1.19, 2.39)	**0.003***	0.086	23 / 38
**Pancreatic**	1.04 (0.83, 1.3)	0.736	0.99 (0.74, 1.32)	0.926	1.12 (0.8, 1.56)	0.52	0.583	52 / 38
**Lung**	1.01 (0.9, 1.14)	0.869	1.04 (0.89, 1.2)	0.645	0.97 (0.81, 1.17)	0.762	0.597	207/ 125
**Hematologic**	1.11 (0.98, 1.25)	0.108	1.04 (0.88, 1.22)	0.655	1.21 (1, 1.46)	**0.045**	0.223	165 / 122
**Melanoma**	0.88 (0.73, 1.07)	0.198	0.87 (0.69, 1.1)	0.246	0.91 (0.66, 1.25)	0.546	0.848	80 / 40
**Breast**	0.93 (0.85, 1.02)	0.139	0.93 (0.85, 1.02)	0.139				635
**Breast (Pre)**			1.18 (0.81, 1.71)	0.386				31
**Breast(Post)**			0.92 (0.84, 1.01)	0.067				600
**Endometrial**	0.98 (0.8, 1.21)	0.866	0.98 (0.8, 1.21)	0.866				108
**Ovarian**	1.05 (0.81, 1.37)	0.701	1.05 (0.81, 1.37)	0.701				80
**Prostate**	0.96 (0.87, 1.07)	0.469			0.96 (0.87, 1.07)	0.469		469
**Kidney**	0.81 (0.64, 1.02)	0.079	0.92 (0.67, 1.25)	0.583	0.7 (0.49, 1)	0.051	0.27	45 / 33
**Bladder**	0.91 (0.68, 1.21)	0.517	1.22 (0.8, 1.88)	0.357	0.75 (0.51, 1.1)	0.138	0.092	25 / 28
**Brain**	0.91 (0.67, 1.24)	0.546	1.06 (0.72, 1.56)	0.764	0.71 (0.42, 1.2)	0.206	0.229	30 / 15
**Overall**	0.99 (0.96, 1.02)	0.38	0.96 (0.93, 1)	0.066	1.02 (0.97, 1.07)	0.391	0.064	3034 / 2022

Model 1: Adjusted for age, gender (female, male), BMI, Townsend deprivation index (TDI), smoking status (never/ former/current), alcohol frequency intake (never, 1-3 t/m, 1-2 t/w, >3 t/w), MET, family history of (No/Yes). **Model 2:** As model 1, plus 9-item dietary intake score, sedentary behaviour, cardiovascular disease (no/yes), diabetes (no/yes), NSAID (no/yes). For prostate cancer, we also adjusted for testosterone and SHBG concentrations. For breast cancer, we also adjusted for history of mammography (no/yes). Additional adjustments for female specific cancers were menopausal status (no/yes), oral contraceptive use (no/yes), HRT use (no/yes), and age at menarche. Pre: pre-menopausal, Post: post-menopausal.

*: FDR-significance

Supplementary Table 6. Sensitivity analysis: Association between individuals with probable sarcopenia versus without sarcopenia for total and site-specific cancer incidence, overall and by sex after excluding 9,230 participants (F=4,335, M=4,895) who experienced any cancer type or death within the first 2 years of follow-up. Data are presented as hazard ratios and 95% confidence intervals. Abbreviations: FDR, False Discovery Rate; HR, Hazard Ratio; CI, Confidence Interval

Cancer	HR(95%CI)	P-Value	Female HR(95%CI)	P-Value	Male HR(95%CI)	P-Value	Interaction by sex P-Value
**Oral**	0.95 (0.69, 1.31)	0.771	0.83 (0.5, 1.37)	0.467	1.06 (0.7, 1.59)	0.794	0.464
**Oesophageal**	1.08 (0.83, 1.41)	0.573	0.83 (0.52, 1.32)	0.419	1.25 (0.91, 1.73)	0.168	0.145
**Gastric**	0.96 (0.68, 1.35)	0.815	0.89 (0.53, 1.48)	0.652	1.02 (0.65, 1.6)	0.921	0.685
**Colorectal**	1.09 (0.97, 1.22)	0.171	0.96 (0.82, 1.13)	0.633	1.26 (1.06, 1.49)	**0.007**	**0.022**
**Liver**	1.37 (1.02, 1.83)	**0.036**	0.99 (0.61, 1.6)	0.969	1.7 (1.18, 2.43)	**0.004**	0.074
**Pancreatic**	1.07 (0.85, 1.35)	0.555	0.98 (0.72, 1.33)	0.884	1.22 (0.86, 1.72)	0.266	0.352
**Lung**	1.04 (0.92, 1.17)	0.579	1.09 (0.93, 1.27)	0.286	0.96 (0.78, 1.17)	0.664	0.315
**Hematologic**	1.1 (0.97, 1.26)	0.152	1.05 (0.88, 1.25)	0.58	1.18 (0.96, 1.44)	0.109	0.39
**Melanoma**	0.9 (0.73, 1.1)	0.3	0.89 (0.7, 1.14)	0.37	0.91 (0.65, 1.28)	0.591	0.927
**Breast**	0.94 (0.84, 1.04)	0.201	0.94 (0.84, 1.04)	0.201			
**Breast (Pre)**			1.01 (0.63, 1.63)	0.965			
**Breast(Post)**			0.93 (0.84, 1.02)	0.114			
**Endometrial**	1.01 (0.8, 1.26)	0.949	1.01 (0.8, 1.26)	0.949			
**Ovarian**	1.08 (0.82, 1.44)	0.574	1.08 (0.82, 1.44)	0.574			
**Prostate**	0.99 (0.89, 1.1)	0.87			0.99 (0.89, 1.1)	0.87	
**Kidney**	0.84 (0.65, 1.07)	0.152	0.97 (0.7, 1.35)	0.862	0.7 (0.48, 1.02)	0.063	0.192
**Bladder**	0.89 (0.65, 1.21)	0.457	1.19 (0.74, 1.92)	0.477	0.74 (0.49, 1.12)	0.154	0.139
**Brain**	0.88 (0.63, 1.24)	0.464	1.03 (0.68, 1.57)	0.889	0.68 (0.38, 1.22)	0.199	0.259
**Overall**	1 (0.97, 1.03)	0.895	0.97 (0.93, 1.01)	0.183	1.04 (0.99, 1.09)	0.156	0.051

Adjusted for age, gender (female, male), BMI, Townsend deprivation index (TDI), smoking status (never/ former/current), alcohol frequency intake (never, 1-3 t/m, 1-2 t/w, >3 t/w), MET, family history of (No/Yes), 9-item dietary intake, sedentary behavior, cardiovascular disease (no/yes), diabetes (no/yes), NSAID (no/yes). For Prostate we also adjusted for testosterone and SHBG. For breast cancer, we also adjusted for history of mammography (no/yes). Additional adjustments for female specific cancers were menopause status (no/yes), contraceptive use (no/yes), HRT (no/yes), and age at menarche. Pre: pre-menopausal, Post: post-menopausal.

*: FDR-significance.

Supplementary Table 7. Linear association for continuous grip strength with total and site-specific cancer incidence, overall and by sex. Data are presented as hazard ratios and 95% confidence intervals. Abbreviations: FDR, False Discovery Rate; HR, Hazard Ratio; CI, Confidence Interval

**Cancer**	**HR(95%CI)**	**P-Value**	**Female HR(95%CI)**	**P-Value**	**Male HR(95%CI)**	**P-Value**	**Interaction by sex P-Value**
**Model 1**							
**Oral**	1 (0.92, 1.08)	0.971	1.1 (0.96, 1.26)	0.188	0.95 (0.86, 1.05)	0.337	0.094
**Oesophageal**	0.99 (0.92, 1.07)	0.85	1.12 (0.97, 1.29)	0.133	0.95 (0.87, 1.04)	0.25	0.054
**Gastric**	0.97 (0.88, 1.07)	0.542	1.08 (0.92, 1.27)	0.359	0.92 (0.82, 1.03)	0.161	0.11
**Colorectal**	1 (0.96, 1.03)	0.827	1.03 (0.98, 1.08)	0.286	0.97 (0.93, 1.02)	0.21	0.094
**Liver**	0.84 (0.76, 0.93)	**0.001***	0.97 (0.82, 1.13)	0.678	0.78 (0.69, 0.88)	**<0.001***	**0.029**
**Pancreatic**	0.97 (0.9, 1.04)	0.388	0.98 (0.89, 1.08)	0.702	0.96 (0.87, 1.06)	0.393	0.751
**Lung**	0.95 (0.92, 0.99)	**0.022**	0.94 (0.89, 0.99)	**0.017**	0.97 (0.92, 1.03)	0.365	0.296
**Hematologic**	0.97 (0.93, 1.01)	0.119	0.96 (0.91, 1.02)	0.195	0.97 (0.92, 1.03)	0.328	0.78
**Melanoma**	1.07 (1.02, 1.13)	**0.007**	1.07 (1, 1.15)	**0.048**	1.08 (1, 1.16)	**0.048**	0.924
**Breast**	1.03 (1, 1.05)	**0.039**	1.03 (1, 1.05)	**0.039**			
**Endometrial**	1.04 (0.97, 1.11)	0.253	1.04 (0.97, 1.11)	0.253			
**Ovarian**	0.98 (0.9, 1.06)	0.617	0.98 (0.9, 1.06)	0.617			
**Prostate**	1.02 (1, 1.05)	0.109			1.02 (1, 1.05)	0.109	
**Kidney**	1.05 (0.98, 1.12)	0.175	1.06 (0.95, 1.17)	0.305	1.04 (0.96, 1.13)	0.336	0.83
**Bladder**	0.94 (0.87, 1.02)	0.165	0.87 (0.74, 1.02)	0.082	0.97 (0.88, 1.07)	0.529	0.237
**Brain**	0.96 (0.88, 1.04)	0.324	0.89 (0.79, 1.01)	0.071	1.01 (0.91, 1.13)	0.816	0.123
**Overall**	1.01 (1, 1.02)	0.051	1.04 (1.03, 1.05)	**<0.001***	0.98 (0.97, 0.99)	**0.004***	**<0.001***
**Model 2**							
**Oral**	1 (0.92, 1.09)	0.977	1.1 (0.96, 1.26)	0.192	0.96 (0.87, 1.06)	0.385	0.107
**Oesophageal**	1 (0.93, 1.08)	0.98	1.12 (0.97, 1.3)	0.114	0.96 (0.87, 1.05)	0.347	0.06
**Gastric**	0.98 (0.88, 1.07)	0.614	1.08 (0.92, 1.27)	0.343	0.93 (0.82, 1.04)	0.197	0.119
**Colorectal**	1 (0.97, 1.04)	0.956	1.03 (0.98, 1.08)	0.238	0.98 (0.93, 1.02)	0.327	0.116
**Liver**	0.88 (0.8, 0.97)	**0.011**	0.99 (0.84, 1.16)	0.857	0.82 (0.73, 0.93)	**0.002***	0.072
**Pancreatic**	0.98 (0.91, 1.05)	0.55	0.99 (0.89, 1.09)	0.775	0.97 (0.88, 1.07)	0.568	0.849
**Lung**	0.98 (0.94, 1.01)	0.212	0.96 (0.91, 1.01)	0.111	0.99 (0.94, 1.05)	0.839	0.32
**Hematologic**	0.97 (0.93, 1.01)	0.146	0.97 (0.91, 1.02)	0.22	0.98 (0.93, 1.03)	0.37	0.778
**Melanoma**	1.07 (1.02, 1.13)	**0.007**	1.07 (1, 1.15)	0.058	1.08 (1, 1.16)	**0.041**	0.838
**Breast**	1.03 (1, 1.06)	0.069	1.03 (1, 1.06)	0.069			
**Breast (Pre)**			1.02 (0.95, 1.1)	0.532			
**Breast(Post)**			1.03 (1.01, 1.06)	**0.021**			
**Endometrial**	1.03 (0.96, 1.1)	0.411	1.03 (0.96, 1.1)	0.411			
**Ovarian**	1 (0.92, 1.1)	0.956	1 (0.92, 1.1)	0.956			
**Prostate**	1.01 (0.99, 1.04)	0.306			1.01 (0.99, 1.04)	0.306	
**Kidney**	1.06 (0.99, 1.13)	0.096	1.06 (0.96, 1.18)	0.242	1.05 (0.97, 1.14)	0.21	0.887
**Bladder**	0.95 (0.88, 1.04)	0.278	0.88 (0.75, 1.03)	0.123	0.98 (0.89, 1.08)	0.698	0.254
**Brain**	0.95 (0.87, 1.04)	0.244	0.89 (0.79, 1.01)	0.076	1 (0.89, 1.12)	0.972	0.183
**Overall**	1.01 (1, 1.02)	**0.008**	1.04 (1.03, 1.05)	**<0.001***	0.99 (0.97, 1)	**0.026**	**<0.001***

Model 1: Adjusted for age, gender (female, male), BMI, Townsend deprivation index (TDI), smoking status (never/ former/current), alcohol frequency intake (never, 1-3 t/m, 1-2 t/w, >3 t/w), MET, family history of (No/Yes). **Model 2:** As model 1, plus 9-item dietary intake score, sedentary behaviour, cardiovascular disease (no/yes), diabetes (no/yes), NSAID (no/yes). For prostate cancer, we also adjusted for testosterone and SHBG concentrations. For breast cancer, we also adjusted for history of mammography (no/yes). Additional adjustments for female specific cancers were menopausal status (no/yes), oral contraceptive use (no/yes), HRT use (no/yes), and age at menarche. Pre: pre-menopausal, Post: post-menopausal

*: FDR-significance

Supplementary Table 8. Association between low muscle mass index versus normal muscle mass index for total and site-specific cancer incidence, overall and by sex. Data are presented as hazard ratios and 95% confidence intervals. Abbreviations: FDR, False Discovery Rate; HR, Hazard Ratio; CI, Confidence Interval

Cancer	HR(95%CI)	P-Value	Female HR(95%CI)	P-Value	Male HR(95%CI)	P-Value	Interaction by sex P-Value	N of Cases (F / M)
**Model 1**								
**Oral**	2.57 (1.81, 3.65)	**<0.001***			2.86 (2, 4.09)	**<0.001***		1 / 46
**Oesophageal**	1.84 (1.23, 2.75)	**0.003***	8.66 (4.51, 16.64)	**<0.001***	1.27 (0.78, 2.07)	0.338	**<0.001***	10 / 18
**Colorectal**	1.07 (0.85, 1.35)	0.583	1.23 (0.73, 2.09)	0.442	1.04 (0.8, 1.34)	0.785	0.563	15 / 71
**Pancreatic**	0.69 (0.38, 1.27)	0.233						3 / 9
**Lung**	1.19 (0.97, 1.45)	0.09	1.37 (0.91, 2.06)	0.136	1.15 (0.92, 1.44)	0.224	0.456	25 / 116
**Hematologic**	0.84 (0.63, 1.13)	0.252			0.85 (0.62, 1.17)	0.317		8 / 51
**Melanoma**	0.88 (0.59, 1.33)	0.557			0.82 (0.5, 1.33)	0.417		7 / 21
**Breast**	0.89 (0.65, 1.21)	0.448	0.89 (0.65, 1.21)	0.448				42
**Prostate**	0.82 (0.71, 0.94)	**0.004***			0.82 (0.71, 0.94)	**0.004***		262
**Kidney**	0.77 (0.43, 1.38)	0.38			0.8 (0.44, 1.47)	0.47		1 / 11
**Bladder**	1.46 (0.95, 2.25)	0.085			1.44 (0.92, 2.25)	0.113		2 / 27
**Brain**	0.64 (0.31, 1.31)	0.223						2 / 9
**Overall**	1.04 (0.98, 1.11)	0.149	0.98 (0.85, 1.13)	0.788	1.06 (0.99, 1.13)	0.085	0.33	228 / 1164
**Model 2**								
**Oral**	2.48 (1.74, 3.54)	**<0.001***			2.84 (1.98, 4.06)	**<0.001***		0 / 40
**Oesophageal**	1.78 (1.19, 2.67)	**0.005***	8.21 (4.27, 15.78)	**<0.001***	1.23 (0.76, 2.01)	0.4	**<0.001***	10 / 18
**Colorectal**	1.02 (0.8, 1.29)	0.886	1.22 (0.72, 2.07)	0.468	0.98 (0.75, 1.27)	0.877	0.468	14 / 60
**Pancreatic**	0.69 (0.38, 1.27)	0.238						3 / 8
**Lung**	1.12 (0.91, 1.37)	0.276	1.09 (0.71, 1.67)	0.693	1.13 (0.9, 1.41)	0.296	0.894	22 / 94
**Hematologic**	0.85 (0.63, 1.13)	0.26			0.85 (0.62, 1.17)	0.328		8 / 41
**Melanoma**	0.9 (0.59, 1.35)	0.605			0.83 (0.51, 1.35)	0.451		7 / 17
**Breast**	0.98 (0.7, 1.37)	0.908	0.98 (0.7, 1.37)	0.908				39
**Breast (Pre)**			1.18 (0.49, 2.87)	0.713				10
**Breast(Post)**			0.88 (0.62, 1.24)	0.451				27
**Prostate**	0.86 (0.74, 1)	0.058			0.86 (0.74, 1)	0.058		229
**Kidney**	0.77 (0.43, 1.37)	0.371			0.8 (0.44, 1.46)	0.468		1 / 11
**Bladder**	1.42 (0.91, 2.21)	0.12			1.39 (0.88, 2.2)	0.159		2 / 21
**Overall**	1.03 (0.97, 1.1)	0.266	0.96 (0.83, 1.1)	0.537	1.05 (0.99, 1.12)	0.129	0.228	202 / 1024

Model 1: Adjusted for age, gender (female, male), BMI, Townsend deprivation index (TDI), smoking status (never/ former/current), alcohol frequency intake (never, 1-3 t/m, 1-2 t/w, >3 t/w), MET, family history of (No/Yes). **Model 2:** As model 1, plus 9-item dietary intake score, sedentary behaviour, cardiovascular disease (no/yes), diabetes (no/yes), NSAID (no/yes). For prostate cancer, we also adjusted for testosterone and SHBG concentrations. For breast cancer, we also adjusted for history of mammography (no/yes). Additional adjustments for female specific cancers were menopausal status (no/yes), oral contraceptive use (no/yes), HRT use (no/yes), and age at menarche. Pre: pre-menopausal, Post: post-menopausal

Cancer types with less than 10 cancer cases were not included in the analyses.

*: FDR-significance

Supplementary Table 9. Sensitivity analysis: Association between low muscle mass index versus normal muscle mass index for total and site-specific cancer incidence, overall and by sex after excluding 9,230 participants (F=4,335, M=4,895) who experienced any cancer type or death within the first 2 years of follow-up. Data are presented as hazard ratios and 95% confidence intervals. Abbreviations: FDR, False Discovery Rate; HR, Hazard Ratio; CI, Confidence Interval

Cancer	HR(95%CI)	P-Value	Female HR(95%CI)	P-Value	Male HR(95%CI)	P-Value	Interaction by sex P-Value
**Oral**	2.73 (1.88, 3.95)	**<0.001***			3.15 (2.16, 4.57)	**<0.001***	
**Oesophageal**	1.83 (1.19, 2.83)	**0.006**	8.45 (4.24, 16.85)	**<0.001***	1.24 (0.73, 2.11)	0.431	**<0.001***
**Colorectal**	1.08 (0.84, 1.39)	0.569	1.22 (0.69, 2.15)	0.503	1.05 (0.79, 1.39)	0.739	0.645
**Pancreatic**	0.58 (0.29, 1.19)	0.137					
**Lung**	1.07 (0.86, 1.33)	0.555	1.11 (0.71, 1.74)	0.649	1.06 (0.83, 1.35)	0.654	0.849
**Hematologic**	0.89 (0.66, 1.21)	0.467			0.89 (0.64, 1.24)	0.485	
**Melanoma**	0.77 (0.48, 1.23)	0.273			0.72 (0.41, 1.26)	0.247	
**Breast**	1.06 (0.75, 1.5)	0.727	1.06 (0.75, 1.5)	0.727			
**Breast (Pre)**			1.46 (0.60, 3.58)	0.405			
**Breast(Post)**			0.93 (0.65, 1.33)	0.677			
**Prostate**	0.81 (0.69, 0.96)	**0.014**			0.81 (0.69, 0.96)	**0.014**	
**Kidney**	0.8 (0.44, 1.47)	0.48			0.83 (0.44, 1.57)	0.569	
**Bladder**	1.37 (0.84, 2.22)	0.203			1.39 (0.85, 2.28)	0.194	
**Brain**	0.8 (0.39, 1.64)	0.542					
**Overall**	1.01 (0.95, 1.08)	0.706	0.98 (0.85, 1.14)	0.823	1.02 (0.95, 1.09)	0.602	0.668

Adjusted for age, gender (female, male), BMI, Townsend deprivation index (TDI), smoking status (never/ former/current), alcohol frequency intake (never, 1-3 t/m, 1-2 t/w, >3 t/w), MET, family history of (No/Yes), 9-item dietary intake, sedentary behavior, cardiovascular disease (no/yes), diabetes (no/yes), NSAID (no/yes). For Prostate we also adjusted for testosterone and SHBG. For breast cancer, we also adjusted for history of mammography (no/yes). Additional adjustments for female specific cancers were menopause status (no/yes), contraceptive use (no/yes), HRT (no/yes), and age at menarche. Pre: pre-menopausal, Post: post-menopausal.

Cancer types with less than 10 cancer cases were not included in the analyses.

*: FDR-significance.

Supplementary Table 10. Linear association index for continuous muscle mass index (MMI) with total and site-specific cancer incidence, overall and by sex. Data are presented as hazard ratios and 95% confidence intervals. Abbreviations: FDR, False Discovery Rate; HR, Hazard Ratio; CI, Confidence Interval

Cancer	HR(95%CI)	P-Value	Female HR(95%CI)	P-Value	Male HR(95%CI)	P-Value	Interaction by sex P-Value
**Model 1**							
**Oral**	0.89 (0.72, 1.09)	0.241	0.91 (0.71, 1.17)	0.458	0.88 (0.72, 1.08)	0.229	0.736
**Oesophageal**	0.84 (0.69, 1.01)	0.057	0.55 (0.43, 0.7)	**<0.001***	0.86 (0.71, 1.03)	0.109	**<0.001***
**Gastric**	1.13 (0.91, 1.41)	0.267	1.17 (0.9, 1.52)	0.231	1.12 (0.9, 1.4)	0.298	0.627
**Colorectal**	0.99 (0.91, 1.07)	0.74	0.92 (0.84, 1.01)	0.089	1.01 (0.93, 1.09)	0.858	**0.006***
**Liver**	0.8 (0.63, 1)	0.053	0.58 (0.43, 0.77)	**<0.001***	0.83 (0.66, 1.05)	0.117	**<0.001***
**Pancreatic**	1.01 (0.86, 1.18)	0.949	0.95 (0.79, 1.14)	0.55	1.03 (0.87, 1.21)	0.745	0.189
**Lung**	0.77 (0.7, 0.84)	**<0.001***	0.74 (0.66, 0.83)	**<0.001***	0.78 (0.71, 0.86)	**<0.001***	0.201
**Hematologic**	1.22 (1.12, 1.33)	**<0.001***	1.22 (1.1, 1.35)	**<0.001***	1.22 (1.12, 1.34)	**<0.001***	0.918
**Melanoma**	1.24 (1.1, 1.39)	**<0.001***	1.19 (1.04, 1.36)	**0.009***	1.27 (1.12, 1.43)	**<0.001***	0.219
**Breast**	1.02 (0.96, 1.08)	0.49	1.02 (0.96, 1.08)	0.49			
**Endometrial**	1.11 (0.98, 1.26)	0.111	1.11 (0.98, 1.26)	0.111			
**Ovarian**	0.9 (0.75, 1.08)	0.242	0.9 (0.75, 1.08)	0.242			
**Prostate**	1.13 (1.06, 1.2)	**<0.001***			1.13 (1.06, 1.2)	**<0.001***	
**Kidney**	1.05 (0.91, 1.22)	0.475	1.04 (0.88, 1.24)	0.622	1.06 (0.91, 1.23)	0.461	0.824
**Bladder**	0.99 (0.81, 1.2)	0.888	0.98 (0.76, 1.26)	0.896	0.99 (0.81, 1.2)	0.89	0.974
**Brain**	0.88 (0.72, 1.08)	0.223	0.8 (0.63, 1.03)	0.079	0.9 (0.73, 1.11)	0.339	0.17
**Overall**	0.99 (0.97, 1.01)	0.562	1.03 (1.01, 1.06)	**0.013***	0.98 (0.96, 1)	0.058	**<0.001***
**Model 2**							
**Oral**	0.9 (0.74, 1.11)	0.323	0.94 (0.73, 1.22)	0.657	0.9 (0.73, 1.1)	0.295	0.552
**Oesophageal**	0.85 (0.7, 1.02)	0.082	0.56 (0.44, 0.72)	**<0.001***	0.87 (0.72, 1.05)	0.147	**<0.001***
**Gastric**	1.16 (0.93, 1.44)	0.182	1.21 (0.93, 1.57)	0.147	1.15 (0.92, 1.43)	0.212	0.556
**Colorectal**	0.99 (0.92, 1.07)	0.844	0.93 (0.84, 1.02)	0.104	1.01 (0.93, 1.1)	0.752	**0.005***
**Liver**	0.81 (0.65, 1.02)	0.075	0.61 (0.46, 0.81)	**0.001***	0.84 (0.67, 1.06)	0.146	**<0.001***
**Pancreatic**	1.01 (0.86, 1.18)	0.936	0.96 (0.8, 1.16)	0.67	1.02 (0.87, 1.21)	0.782	0.319
**Lung**	0.78 (0.71, 0.86)	**<0.001***	0.77 (0.69, 0.86)	**<0.001***	0.79 (0.72, 0.87)	**<0.001***	0.412
**Hematologic**	1.23 (1.12, 1.34)	**<0.001***	1.22 (1.11, 1.36)	**<0.001***	1.23 (1.12, 1.35)	**<0.001***	0.9
**Melanoma**	1.25 (1.11, 1.4)	**<0.001***	1.2 (1.05, 1.37)	**0.008***	1.28 (1.13, 1.44)	**<0.001***	0.208
**Breast**	1 (0.94, 1.07)	0.99	1 (0.94, 1.07)	0.99			
**Breast (Pre)**			0.90 (0.76, 1.07)	0.236			
**Breast(Post)**			1.03 (0.96, 1.09)	0.411			
**Endometrial**	1.07 (0.94, 1.22)	0.323	1.07 (0.94, 1.22)	0.323			
**Ovarian**	0.86 (0.7, 1.05)	0.134	0.86 (0.7, 1.05)	0.134			
**Prostate**	1.13 (1.06, 1.21)	**<0.001***			1.13 (1.06, 1.21)	**<0.001***	
**Kidney**	1.06 (0.91, 1.23)	0.45	1.06 (0.89, 1.26)	0.529	1.06 (0.91, 1.23)	0.452	0.977
**Bladder**	0.97 (0.8, 1.18)	0.775	0.98 (0.76, 1.26)	0.872	0.97 (0.8, 1.18)	0.77	0.921
**Brain**	0.87 (0.7, 1.07)	0.178	0.79 (0.62, 1.01)	0.06	0.89 (0.72, 1.1)	0.281	0.161
**Overall**	1 (0.98, 1.02)	0.789	1.04 (1.01, 1.06)	**0.004***	0.98 (0.96, 1)	0.107	**<0.001***

Model 1: Adjusted for age, gender (female, male), BMI, Townsend deprivation index (TDI), smoking status (never/ former/current), alcohol frequency intake (never, 1-3 t/m, 1-2 t/w, >3 t/w), MET, family history of (No/Yes). **Model 2:** As model 1, plus 9-item dietary intake score, sedentary behaviour, cardiovascular disease (no/yes), diabetes (no/yes), NSAID (no/yes). For prostate cancer, we also adjusted for testosterone and SHBG concentrations. For breast cancer, we also adjusted for history of mammography (no/yes). Additional adjustments for female specific cancers were menopausal status (no/yes), oral contraceptive use (no/yes), HRT use (no/yes), and age at menarche. Pre: pre-menopausal, Post: post-menopausal

*: FDR-significance

Supplementary Table 11. Association between slow walking pace versus normal walking pace for total and site-specific cancer incidence, overall and by sex. Data are presented as hazard ratios and 95% confidence intervals. Abbreviations: FDR, False Discovery Rate; HR, Hazard Ratio; CI, Confidence Interval

Cancer	HR(95%CI)	P-Value	Female HR(95%CI)	P-Value	Male HR(95%CI)	P-Value	Interaction by sex P-Value	N of Cases (F / M)
**Model 1**								
**Oral**	1.35 (1.04, 1.76)	**0.022**	1.44 (0.93, 2.23)	0.1	1.31 (0.96, 1.79)	0.084	0.725	27 / 55
**Oesophageal**	0.97 (0.76, 1.24)	0.812	0.83 (0.52, 1.33)	0.443	1.03 (0.77, 1.36)	0.861	0.442	21 / 68
**Gastric**	0.99 (0.73, 1.35)	0.952	0.9 (0.53, 1.54)	0.71	1.03 (0.72, 1.49)	0.857	0.673	16 / 42
**Colorectal**	0.98 (0.87, 1.1)	0.703	0.86 (0.72, 1.03)	0.106	1.07 (0.92, 1.25)	0.354	0.059	156 / 228
**Liver**	1.52 (1.15, 2.01)	**0.003***	1.03 (0.64, 1.66)	0.91	1.86 (1.35, 2.56)	**<0.001***	**0.036**	23 / 61
**Pancreatic**	1.14 (0.91, 1.42)	0.264	0.99 (0.71, 1.38)	0.951	1.27 (0.95, 1.7)	0.1	0.241	47 / 64
**Lung**	1.46 (1.32, 1.62)	**<0.001***	1.58 (1.37, 1.82)	**<0.001***	1.36 (1.18, 1.56)	**<0.001***	0.122	279 / 314
**Hematologic**	0.99 (0.87, 1.14)	0.931	0.96 (0.79, 1.18)	0.721	1.02 (0.85, 1.23)	0.829	0.675	119 / 157
**Melanoma**	0.77 (0.62, 0.96)	**0.021**	0.74 (0.55, 0.99)	**0.042**	0.82 (0.6, 1.12)	0.215	0.626	55 / 47
**Breast**	1 (0.91, 1.1)	0.981	1 (0.91, 1.1)	0.981				565
**Endometrial**	0.98 (0.8, 1.2)	0.841	0.98 (0.8, 1.2)	0.841				136
**Ovarian**	1.06 (0.8, 1.42)	0.675	1.06 (0.8, 1.42)	0.675				67
**Prostate**	0.82 (0.75, 0.91)	**<0.001***			0.82 (0.75, 0.91)	**<0.001***		515
**Kidney**	1.07 (0.87, 1.32)	0.516	1.15 (0.85, 1.57)	0.371	1.02 (0.78, 1.33)	0.887	0.544	52 / 74
**Bladder**	1.06 (0.81, 1.37)	0.68	1.4 (0.89, 2.2)	0.145	0.95 (0.7, 1.29)	0.741	0.152	30 / 63
**Brain**	0.79 (0.56, 1.12)	0.189	0.7 (0.41, 1.2)	0.197	0.86 (0.56, 1.35)	0.52	0.554	19 / 26
**Overall**	1.06 (1.03, 1.09)	**<0.001***	1.1 (1.05, 1.15)	**<0.001***	1.02 (0.98, 1.07)	0.315	**0.013**	2609 / 2817
**Model 2**								
**Oral**	1.33 (1.02, 1.74)	**0.033**	1.43 (0.92, 2.21)	0.112	1.29 (0.94, 1.77)	0.112	0.707	24 / 50
**Oesophageal**	0.93 (0.73, 1.2)	0.597	0.8 (0.5, 1.29)	0.362	0.99 (0.74, 1.31)	0.932	0.446	20 / 62
**Gastric**	0.99 (0.72, 1.36)	0.954	0.9 (0.53, 1.53)	0.7	1.04 (0.72, 1.5)	0.846	0.658	16 / 36
**Colorectal**	0.98 (0.87, 1.11)	0.755	0.86 (0.72, 1.03)	0.104	1.08 (0.93, 1.26)	0.297	**0.048**	140 / 203
**Liver**	1.23 (0.93, 1.63)	0.155	0.87 (0.54, 1.4)	0.566	1.46 (1.05, 2.02)	**0.024**	0.066	21 / 54
**Pancreatic**	1.11 (0.88, 1.39)	0.373	0.99 (0.71, 1.38)	0.953	1.22 (0.91, 1.64)	0.184	0.333	43 / 56
**Lung**	1.29 (1.16, 1.43)	**<0.001***	1.38 (1.2, 1.6)	**<0.001***	1.2 (1.04, 1.38)	**0.012**	0.147	253 / 271
**Hematologic**	0.98 (0.85, 1.12)	0.732	0.95 (0.78, 1.16)	0.618	1 (0.83, 1.2)	0.99	0.714	114 / 131
**Melanoma**	0.77 (0.61, 0.96)	**0.019**	0.74 (0.55, 0.99)	**0.045**	0.8 (0.58, 1.11)	0.179	0.709	49 / 41
**Breast**	0.97 (0.87, 1.08)	0.592	0.97 (0.87, 1.08)	0.592				528
**Breast (Pre)**			1.02 (0.69, 1.51)	0.934				31
**Breast(Post)**			0.97 (0.88, 1.08)	0.614				490
**Endometrial**	1.03 (0.83, 1.27)	0.808	1.03 (0.83, 1.27)	0.808				131
**Ovarian**	1.1 (0.8, 1.51)	0.558	1.1 (0.8, 1.51)	0.558				57
**Prostate**	0.86 (0.77, 0.96)	**0.006**			0.86 (0.77, 0.96)	**0.006**		450
**Kidney**	1.02 (0.83, 1.26)	0.85	1.11 (0.82, 1.51)	0.504	0.96 (0.74, 1.26)	0.776	0.471	51 / 67
**Bladder**	1.03 (0.79, 1.35)	0.811	1.39 (0.88, 2.19)	0.157	0.92 (0.68, 1.26)	0.614	0.133	23 / 50
**Brain**	0.79 (0.56, 1.13)	0.194	0.7 (0.41, 1.19)	0.187	0.87 (0.56, 1.37)	0.553	0.519	15 / 22
**Overall**	1.04 (1.01, 1.07)	**0.014**	1.08 (1.04, 1.13)	**<0.001***	1 (0.96, 1.04)	0.975	**0.008**	2410 / 2480

Model 1: Adjusted for age, gender (female, male), BMI, Townsend deprivation index (TDI), smoking status (never/ former/current), alcohol frequency intake (never, 1-3 t/m, 1-2 t/w, >3 t/w), MET, family history of (No/Yes). **Model 2:** As model 1, plus 9-item dietary intake score, sedentary behaviour, cardiovascular disease (no/yes), diabetes (no/yes), NSAID (no/yes). For prostate cancer, we also adjusted for testosterone and SHBG concentrations. For breast cancer, we also adjusted for history of mammography (no/yes). Additional adjustments for female specific cancers were menopausal status (no/yes), oral contraceptive use (no/yes), HRT use (no/yes), and age at menarche. Pre: pre-menopausal, Post: post-menopausal

*: FDR-significance

Supplementary Table 12. Sensitivity analysis: Association between slow waking pace versus normal walking pace for total and site-specific cancer incidence, overall and by sex after excluding 9,230 participants (F=4,335, M=4,895) who experienced any cancer type or death within the first 2 years of follow-up. Data are presented as hazard ratios and 95% confidence intervals. Abbreviations: FDR, False Discovery Rate; HR, Hazard Ratio; CI, Confidence Interval

**Cancer**	**HR(95%CI)**	**P-Value**	**Female HR(95%CI)**	**P-Value**	**Male HR(95%CI)**	**P-Value**	**Interaction by sex P-Value**
**Oral**	1.26 (0.95, 1.68)	0.108	1.41 (0.89, 2.23)	0.138	1.19 (0.84, 1.69)	0.32	0.55
**Oesophageal**	0.91 (0.7, 1.2)	0.507	0.89 (0.55, 1.43)	0.624	0.92 (0.67, 1.26)	0.616	0.89
**Gastric**	1.09 (0.78, 1.51)	0.616	1 (0.58, 1.71)	0.999	1.14 (0.77, 1.67)	0.523	0.698
**Colorectal**	0.94 (0.83, 1.07)	0.384	0.83 (0.69, 1.01)	0.063	1.04 (0.88, 1.23)	0.617	0.073
**Liver**	1.19 (0.89, 1.6)	0.24	0.85 (0.51, 1.4)	0.517	1.41 (1, 1.98)	**0.048**	0.085
**Pancreatic**	0.99 (0.77, 1.27)	0.957	0.87 (0.6, 1.25)	0.442	1.11 (0.81, 1.54)	0.509	0.292
**Lung**	1.28 (1.14, 1.43)	**<0.001***	1.35 (1.16, 1.58)	**<0.001***	1.2 (1.03, 1.4)	**0.017**	0.263
**Hematologic**	0.94 (0.81, 1.1)	0.445	0.88 (0.71, 1.09)	0.25	1 (0.82, 1.22)	0.988	0.376
**Melanoma**	0.8 (0.63, 1.02)	0.068	0.81 (0.59, 1.1)	0.172	0.8 (0.56, 1.13)	0.206	0.972
**Breast**	0.98 (0.87, 1.1)	0.71	0.98 (0.87, 1.1)	0.71			
**Breast (Pre)**			0.92 (0.56, 1.51)	0.752			
**Breast(Post)**			0.99 (0.89, 1.11)	0.862			
**Endometrial**	1.04 (0.82, 1.31)	0.749	1.04 (0.82, 1.31)	0.749			
**Ovarian**	1.08 (0.76, 1.52)	0.669	1.08 (0.76, 1.52)	0.669			
**Prostate**	0.87 (0.78, 0.98)	**0.018**			0.87 (0.78, 0.98)	**0.018**	
**Kidney**	1 (0.8, 1.25)	0.989	1.04 (0.74, 1.45)	0.835	0.97 (0.73, 1.29)	0.853	0.771
**Bladder**	0.98 (0.73, 1.31)	0.896	1.3 (0.78, 2.17)	0.311	0.89 (0.63, 1.24)	0.484	0.203
**Brain**	0.76 (0.51, 1.11)	0.157	0.7 (0.4, 1.25)	0.231	0.8 (0.48, 1.33)	0.39	0.735
**Overall**	1.04 (1, 1.07)	**0.032**	1.08 (1.03, 1.13)	**0.001***	1 (0.95, 1.05)	0.985	**0.017**

Adjusted for age, gender (female, male), BMI, Townsend deprivation index (TDI), smoking status (never/ former/current), alcohol frequency intake (never, 1-3 t/m, 1-2 t/w, >3 t/w), MET, family history of (No/Yes), 9-item dietary intake, sedentary behavior, cardiovascular disease (no/yes), diabetes (no/yes), NSAID (no/yes). For Prostate we also adjusted for testosterone and SHBG. For breast cancer, we also adjusted for history of mammography (no/yes). Additional adjustments for female specific cancers were menopause status (no/yes), contraceptive use (no/yes), HRT (no/yes), and age at menarche. Pre: pre-menopausal, Post: post-menopausal.

*: FDR-significance.

Supplementary Table 13. Association between sarcopenic obesity versus non-sarcopenic obesity for total and site-specific cancer incidence, overall and by sex. Data are presented as hazard ratios and 95% confidence intervals. Abbreviations: FDR, False Discovery Rate; HR, Hazard Ratio; CI, Confidence Interval

Cancer	HR(95%CI)	P-Value	Female HR(95%CI)	P-Value	Male HR(95%CI)	P-Value	Interaction by sex P-Value	N of Cases (F / M)
**Model 1**								
**Oral**	0.79 (0.43, 1.43)	0.431			0.89 (0.43, 1.83)	0.745		4 / 10
**Oesophageal**	1.31 (0.88, 1.93)	0.181			1.34 (0.85, 2.1)	0.205		9 / 26
**Gastric**	0.96 (0.57, 1.63)	0.883			0.92 (0.46, 1.83)	0.808		7 / 10
**Colorectal**	1.15 (0.96, 1.38)	0.139	0.99 (0.75, 1.29)	0.919	1.33 (1.04, 1.7)	**0.025**	0.108	68 / 78
**Liver**	1.2 (0.77, 1.86)	0.413			1.33 (0.78, 2.25)	0.298		8 / 21
**Pancreatic**	1.12 (0.78, 1.6)	0.542	0.96 (0.59, 1.57)	0.879	1.34 (0.81, 2.23)	0.26	0.355	22 / 21
**Lung**	1.12 (0.91, 1.39)	0.276	1.2 (0.91, 1.57)	0.191	1.03 (0.74, 1.43)	0.872	0.475	73 / 45
**Hematologic**	1.06 (0.84, 1.32)	0.632	0.96 (0.71, 1.3)	0.776	1.2 (0.86, 1.65)	0.283	0.325	50 / 44
**Melanoma**	1.05 (0.76, 1.44)	0.77	1.05 (0.7, 1.56)	0.827	1.05 (0.64, 1.73)	0.843	0.987	29 / 19
**Breast**	0.94 (0.81, 1.09)	0.402	0.94 (0.81, 1.09)	0.402				218
**Endometrial**	0.85 (0.64, 1.14)	0.28	0.85 (0.64, 1.14)	0.28				61
**Ovarian**	1.06 (0.68, 1.64)	0.791	1.06 (0.68, 1.64)	0.791				27
**Prostate**	0.97 (0.81, 1.16)	0.718			0.97 (0.81, 1.16)	0.718		147
**Kidney**	0.99 (0.71, 1.39)	0.951	1 (0.62, 1.62)	0.984	0.97 (0.61, 1.56)	0.915	0.928	20 / 20
**Bladder**	1.18 (0.77, 1.81)	0.452	2.07 (1.11, 3.84)	**0.022**	0.78 (0.42, 1.45)	0.438	0.029	16 / 13
**Brain**	1.06 (0.62, 1.84)	0.825	1.37 (0.69, 2.72)	0.372				10 / 6
**Overall**	1.01 (0.95, 1.06)	0.847	0.99 (0.92, 1.06)	0.76	1.03 (0.95, 1.12)	0.504	0.477	1016 / 716
**Model 2**								
**Oral**	0.78 (0.43, 1.42)	0.418						4 / 8
**Oesophageal**	1.3 (0.88, 1.93)	0.184			1.34 (0.85, 2.1)	0.205		8 / 22
**Gastric**	0.98 (0.58, 1.67)	0.941						7 / 9
**Colorectal**	1.14 (0.95, 1.37)	0.165	0.98 (0.75, 1.28)	0.87	1.31 (1.03, 1.68)	**0.03**	0.111	62 / 72
**Liver**	1.1 (0.71, 1.71)	0.674			1.18 (0.7, 2.01)	0.533		8 / 16
**Pancreatic**	1.08 (0.75, 1.56)	0.665	0.97 (0.59, 1.58)	0.895	1.25 (0.74, 2.11)	0.406	0.483	19 / 16
**Lung**	1.08 (0.87, 1.33)	0.483	1.14 (0.87, 1.49)	0.358	1 (0.72, 1.39)	0.989	0.56	65 / 41
**Hematologic**	1.06 (0.84, 1.33)	0.623	0.95 (0.7, 1.29)	0.76	1.21 (0.87, 1.67)	0.261	0.299	49 / 41
**Melanoma**	1.05 (0.76, 1.44)	0.779	1.05 (0.7, 1.57)	0.818	1.04 (0.63, 1.72)	0.868	0.987	28 / 18
**Breast**	0.92 (0.78, 1.09)	0.346	0.92 (0.78, 1.09)	0.346				205
**Breast (Pre)**			1.49 (0.69, 3.20)	0.307				12
**Breast(Post)**			0.92 (0.79, 1.08)	0.308				192
**Endometrial**	0.9 (0.68, 1.21)	0.495	0.9 (0.68, 1.21)	0.495				55
**Ovarian**	1.13 (0.7, 1.83)	0.617	1.13 (0.7, 1.83)	0.617				25
**Prostate**	1.03 (0.85, 1.24)	0.774			1.03 (0.85, 1.24)	0.774		133
**Kidney**	0.96 (0.69, 1.35)	0.831	0.98 (0.61, 1.57)	0.919	0.95 (0.59, 1.53)	0.84	0.943	20 / 19
**Bladder**	1.15 (0.75, 1.76)	0.526	2.03 (1.09, 3.78)	**0.026**	0.76 (0.41, 1.41)	0.384	**0.027**	14 / 11
**Brain**	1.08 (0.63, 1.87)	0.772	1.38 (0.69, 2.75)	0.359				10 / 5
**Overall**	1 (0.95, 1.05)	0.948	0.99 (0.92, 1.06)	0.672	1.02 (0.94, 1.1)	0.687	0.558	949 / 642

Model 1: Adjusted for age, gender (female, male), BMI, Townsend deprivation index (TDI), smoking status (never/ former/current), alcohol frequency intake (never, 1-3 t/m, 1-2 t/w, >3 t/w), MET, family history of (No/Yes). **Model 2:** As model 1, plus 9-item dietary intake score, sedentary behaviour, cardiovascular disease (no/yes), diabetes (no/yes), NSAID (no/yes). For prostate cancer, we also adjusted for testosterone and SHBG concentrations. For breast cancer, we also adjusted for history of mammography (no/yes). Additional adjustments for female specific cancers were menopausal status (no/yes), oral contraceptive use (no/yes), HRT use (no/yes), and age at menarche.

Cancer types with less than 10 cancer cases were not included in the analyses. Pre: pre-menopausal, Post: post-menopausal.

*: FDR-significance

Supplementary Table 14. Sensitivity analysis: Association between sarcopenic obesity versus non-sarcopenic obesity for total and site-specific cancer incidence, overall and by sex after excluding 9,230 participants (F=4,335, M=4,895) who experienced any cancer type or death within the first 2 years of follow-up. Data are presented as hazard ratios and 95% confidence intervals. Abbreviations: FDR, False Discovery Rate; HR, Hazard Ratio; CI, Confidence Interval

Cancer	HR(95%CI)	P-Value	Female HR(95%CI)	P-Value	Male HR(95%CI)	P-Value	Interaction by sex P-Value
**Oral**	0.64 (0.32, 1.27)	0.206			0.71 (0.31, 1.64)	0.424	
**Oesophageal**	1.44 (0.96, 2.15)	0.077			1.5 (0.94, 2.39)	0.088	
**Gastric**	1.04 (0.6, 1.79)	0.9			0.97 (0.47, 2.03)	0.939	
**Colorectal**	1.13 (0.93, 1.38)	0.229	0.94 (0.7, 1.26)	0.678	1.34 (1.03, 1.75)	**0.029**	0.072
**Liver**	1.08 (0.68, 1.71)	0.745			1.19 (0.68, 2.05)	0.544	
**Pancreatic**	1.14 (0.78, 1.65)	0.507	1.02 (0.62, 1.7)	0.933	1.29 (0.75, 2.23)	0.35	0.528
**Lung**	1.06 (0.85, 1.33)	0.612	1.09 (0.82, 1.46)	0.554	1.02 (0.72, 1.44)	0.925	0.756
**Hematologic**	1.06 (0.83, 1.34)	0.657	0.95 (0.69, 1.31)	0.748	1.21 (0.86, 1.72)	0.28	0.308
**Melanoma**	1.02 (0.72, 1.43)	0.926	0.97 (0.62, 1.51)	0.889	1.09 (0.64, 1.86)	0.743	0.731
**Breast**	0.94 (0.78, 1.14)	0.547	0.94 (0.78, 1.14)	0.547			
**Breast (Pre)**			1.34 (0.40, 4.46)	0.636			
**Breast(Post)**			0.95 (0.80, 1.12)	0.535			
**Endometrial**	0.9 (0.66, 1.23)	0.519	0.9 (0.66, 1.23)	0.519			
**Ovarian**	1.23 (0.74, 2.05)	0.424	1.23 (0.74, 2.05)	0.424			
**Prostate**	1.03 (0.84, 1.27)	0.742			1.03 (0.84, 1.27)	0.742	
**Kidney**	0.96 (0.67, 1.38)	0.829	1.06 (0.65, 1.74)	0.806	0.87 (0.52, 1.45)	0.59	0.573
**Bladder**	0.96 (0.59, 1.56)	0.878	1.63 (0.8, 3.34)	0.178	0.67 (0.34, 1.33)	0.257	0.077
**Brain**	1.02 (0.56, 1.88)	0.945	1.37 (0.67, 2.83)	0.39			
**Overall**	1 (0.94, 1.06)	0.986	0.98 (0.91, 1.06)	0.658	1.03 (0.94, 1.12)	0.577	0.473

Adjusted for age, gender (female, male), BMI, Townsend deprivation index (TDI), smoking status (never/ former/current), alcohol frequency intake (never, 1-3 t/m, 1-2 t/w, >3 t/w), MET, family history of (No/Yes), 9-item dietary intake, sedentary behavior, cardiovascular disease (no/yes), diabetes (no/yes), NSAID (no/yes). For Prostate we also adjusted for testosterone and SHBG. For breast cancer, we also adjusted for history of mammography (no/yes). Additional adjustments for female specific cancers were menopause status (no/yes), contraceptive use (no/yes), HRT (no/yes), and age at menarche. Pre: pre-menopausal, Post: post-menopausal.

Cancer types with less than 10 cancer cases were not included in the analyses.

*: FDR-significance.

Supplementary Figure

Supplementary Figure 1: Number of cancer cases in UK Biobank recorded from UK registries between 2018 and 2022.

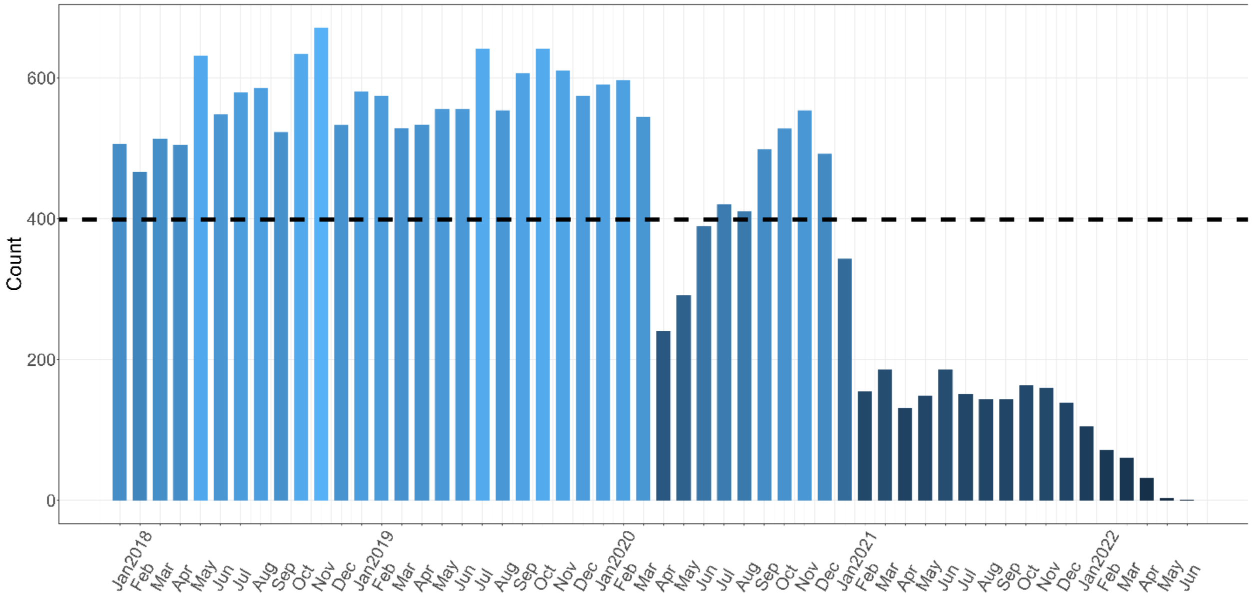

Supplementary Figure 2. Percentages of Covariates missing values.

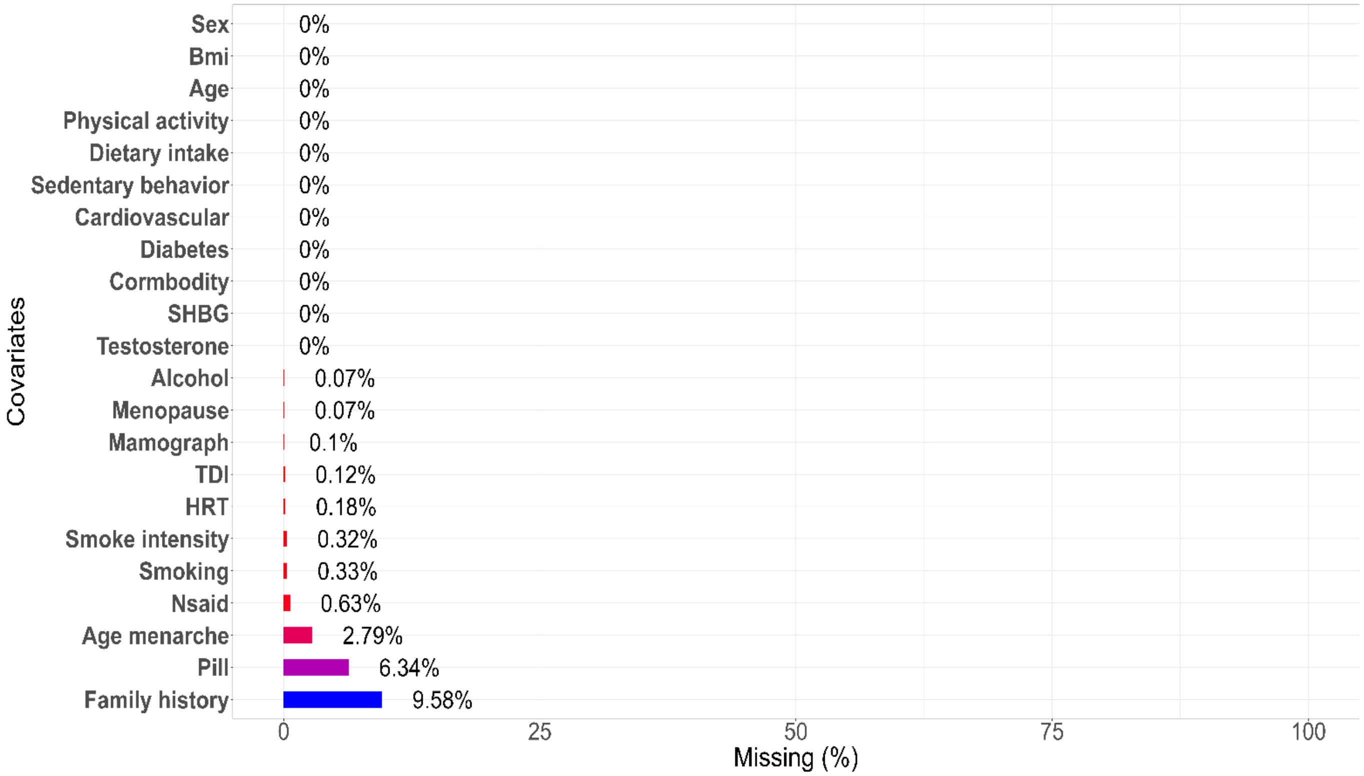

Supplementary Figure 3. Model 2 association of standardized relative grip strength (kg) with cancer risk stratified by sex allowing for nonlinear effects. Reference value is 23.5 (6.16) and 40 (8.69) median (SD) value for females/males, respectively. Separate models were fitted for each cancer type, each with a restricted cubic spline for body composition (knots placed at 5th, 35th, 65th and 95th centiles). Red and blue colors represent females/males respectively.

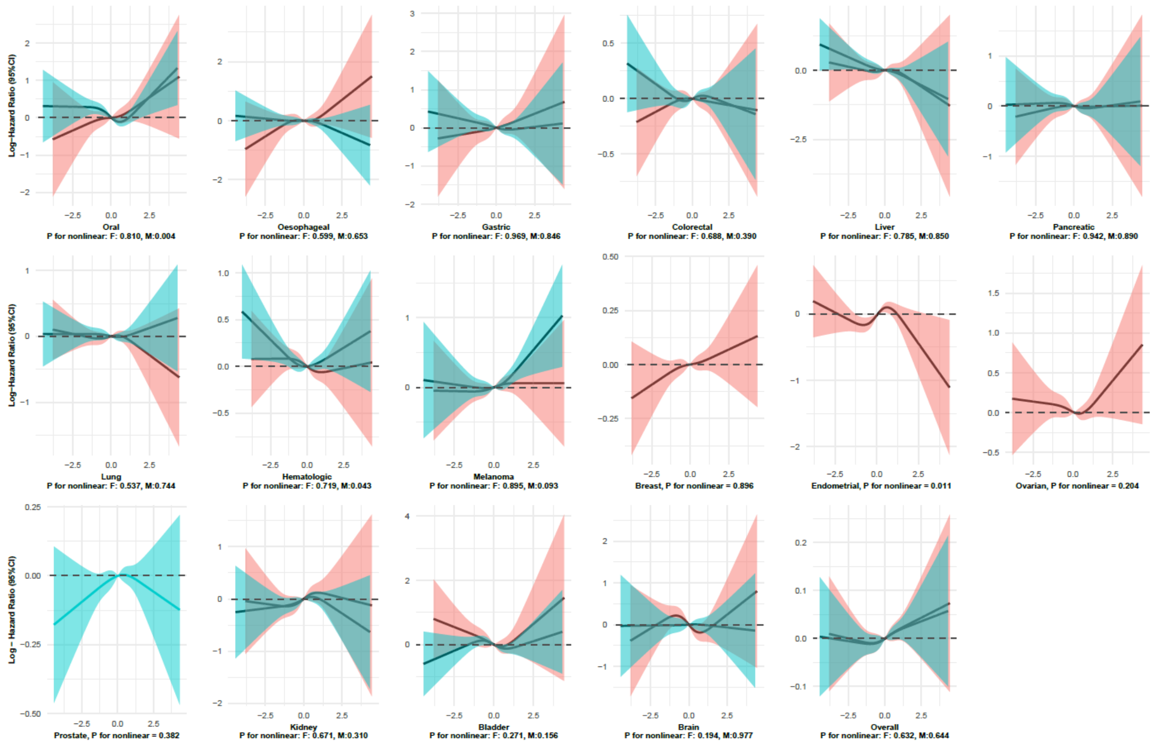

Supplementary Figure 4. Model 2 association of standardized relative muscle mass index (kg) with cancer risk stratified by sex allowing for nonlinear effects. Reference is 6.81 (0.82) and 8.62 (1.05) median (SD) value for females/males, respectively. Separate models were fitted for each cancer type, each with a restricted cubic spline for body composition (knots placed at 5th, 35th, 65th and 95th centiles). Red and blue colours represent females/males respectively.

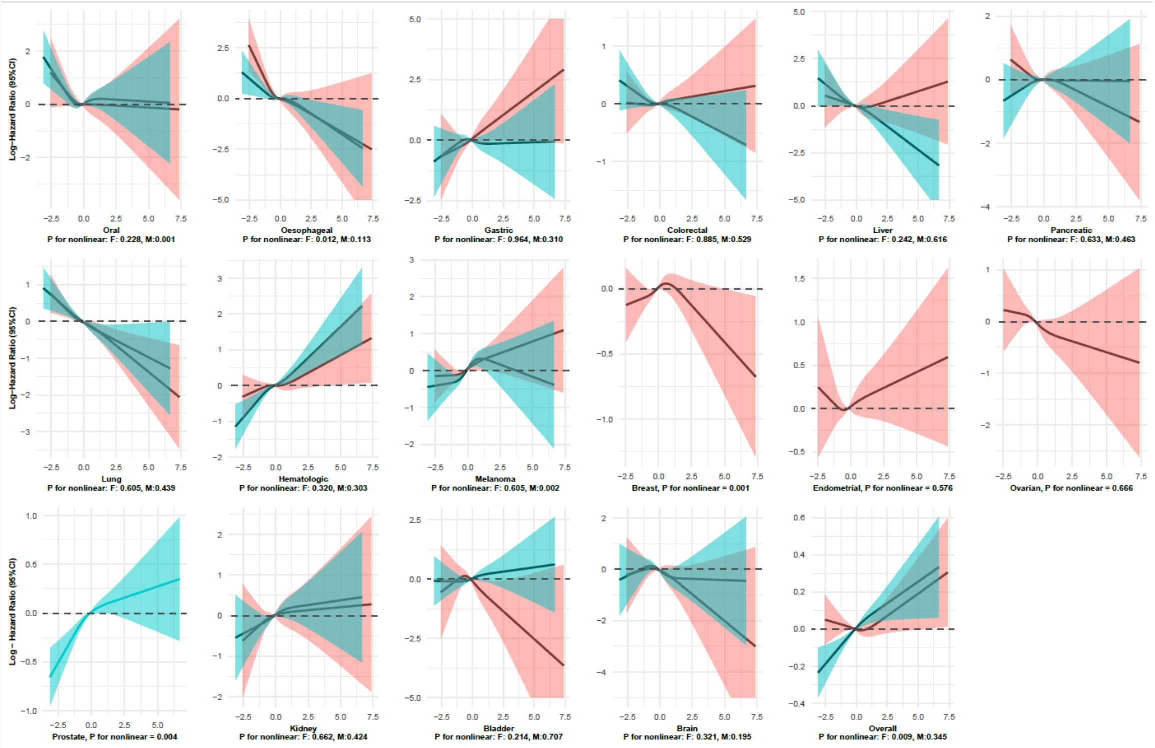

References

1. Petermann-Rocha F, Gray SR, Forrest E, et al. Associations of muscle mass and grip strength with severe NAFLD: A prospective study of 333,295 UK Biobank participants. *J Hepatol* 2022;76(5): 1021-1029.
2. Christakoudi S, Riboli E, Evangelou E, Tsilidis KK. Associations of body shape index (ABSI) and hip index with liver, metabolic, and inflammatory biomarkers in the UK Biobank cohort. *Sci Rep* 2022;12(1): 8812.
3. Health and Deprivation P Townsend P Phillimore A Beattie Health and Deprivation Published by Croom Helm 212pp £19.95 0-7099-4351-2 [Formula: see text]. *Nurs Stand* 1988;2(17): 34.
4. Peng H, Wang S, Wang M, et al. Lifestyle Factors, Genetic Risk, and Cardiovascular Disease Risk among Breast Cancer Survivors: A Prospective Cohort Study in UK Biobank. *Nutrients* 2023;15(4).
5. Nakada S, Ho FK, Celis-Morales C, Pell JP. Association between being breastfed and cardiovascular disease: a population cohort study of 320 249 participants. *J Public Health (Oxf)* 2023;45(3): 569-576.
6. Christakoudi S, Tsilidis KK, Evangelou E, Riboli E. A Body Shape Index (ABSI), hip index, and risk of cancer in the UK Biobank cohort. 2021;10(16): 5614-5628.
7. UK Biobank website: https://www.ukbiobank.ac.uk/ (last accessed 01/10/2023).
8. Data providers and dates of data availability, UK Biobank website: https://biobank.ndph.ox.ac.uk/showcase/exinfo.cgi?src=Data_providers_and_dates (last accessed 10/01/2024).
9. Cruz-Jentoft AJ, Bahat G, Bauer J, et al. Sarcopenia: revised European consensus on definition and diagnosis. *Age Ageing* 2019;48(1): 16-31.
10. Christakoudi S, Kakourou A, Markozannes G, et al. Blood pressure and risk of cancer in the European Prospective Investigation into Cancer and Nutrition. *Int J Cancer* 2020;146(10): 2680-2693.
